# The Role of a Food Literacy Intervention in Promoting Food Security and Food Literacy—OzHarvest’s NEST Program

**DOI:** 10.3390/nu12082197

**Published:** 2020-07-23

**Authors:** Elisha G. West, Rebecca Lindberg, Kylie Ball, Sarah A. McNaughton

**Affiliations:** Institute for Physical Activity and Nutrition, Deakin University, Geelong, VIC 3220, Australia; r.lindberg@deakin.edu.au (R.L.); sarah.mcnaughton@deakin.edu.au (S.A.M.)

**Keywords:** household food security, nutrition education, food literacy, intervention, public health

## Abstract

Food literacy interventions are widely implemented to improve the food security and health of low-socioeconomic adults. The purpose of this study was to conduct an inquiry into the value of OzHarvest’s six-week NEST (Nutrition Education and Skills Training) program in promoting food security and food literacy, and to identify the barriers and enablers that participants experienced in sustaining food security, and in utilising their food literacy skills beyond the program. A descriptive evaluation study with pre-post surveys (*n* = 21) and post-program interviews (*n* = 17) was conducted, with a convenience sample of NEST program participants living in Sydney, Newcastle, and Melbourne, Australia. Participants demonstrated improvements in food security status (*p* = 0.030), cooking confidence (*p* = 0.001), food preparation behaviours (*p* = 0.006), nutrition knowledge (*p* = 0.033), vegetable consumption (*p* = 0.043), and a reduction in intake of sugar-sweetened beverages (*p* = 0.017), and salty snack foods (*p* = 0.011). The interviews revealed that participants learned to stretch their food budgets and make meaningful changes to their food utilisation (a key dimension of food security). Interviews also identified enablers (e.g., social support) and barriers (e.g., health conditions) to achieving food security. Acknowledging the need for a multi-faceted approach that also addresses upstream determinants, interventions like NEST may play a role in promoting food security and food literacy.

## 1. Introduction

Globally, one in five deaths are attributed to a poor diet [1], with obesity, undernutrition, and other diet-related conditions substantially contributing to poor health worldwide [2]. The number of people affected by hunger and food insecurity has increased from 804 million in 2016, to approximately 821 million in 2017 [3], and COVID-19 is predicted to worsen its prevalence and severity. Food insecurity is commonly viewed as an issue for low-income countries; however, it is also a growing public health challenge in high-income countries [4]. While some surveys have reported that food insecurity impacts 5% of Australians [4,5], there are alternative studies that estimate that up to 18% of Australian adults and 22% of Australian children experience this social and health problem [6].

Food insecurity is defined as the ‘limited or uncertain availability of nutritionally adequate and safe foods, or [the] limited or uncertain ability to acquire acceptable food in socially acceptable ways’ [7] (p. 1). The four dimensions that contribute to food security are (1) the physical availability of food; (2) economic, physical, and socio-cultural access to food; (3) food utilisation; (4) stability of these three dimensions over time [7]. Of these dimensions, Begley et al. assert that utilisation has attracted the least amount of research [8]. It covers the physical, social, and human resources required to safely transform food items into meals [8,9]. This dimension includes the food literacy skills, knowledge, and socio-cultural dimensions that impact an individual’s understanding of which food items to select and how to prepare and store them [9].

The term ‘food literacy’ has significantly increased in policy, practice, and research over the past decade [10]. Vidgen and Gallegos define food literacy as ‘a collection of inter-related knowledge, skills and behaviours required to plan, manage, select, prepare and eat food to meet needs and determine intake’ [10] (p. 54), and as, ‘the scaffolding that empowers individuals, households, communities or nations to protect diet quality through change and strengthen dietary resilience over time’ [10] (p. 54). Food literacy interventions have been shown to provide individuals with these skills that contribute to health [10,11]. However, the role that food literacy interventions play in addressing food security remains unclear [8,11]. Poor food literacy may exacerbate food insecurity, or experiencing food insecurity may restrict an individual’s ability to practice food literacy behaviours and achieve adequate diet quality [8]. Hence, food literacy interventions cannot address the upstream determinants of food insecurity, such as access and affordability [8,11]. There remains a strong need for upstream interventions and policies, therefore, food literacy interventions can only be expected to be part of this broader approach to improving food security.

There is evidence to suggest that improved food security and food literacy skills can result from these interventions [12,13,14,15], with some studies showing significant improvements sustained over time, and independent of environmental factors [16,17]. These changes may be due to improved nutrition knowledge and food literacy skills, assisting participants to maximise their income [8]. However, other studies have shown no effects on food security, despite showing effects on food literacy knowledge and skills [18,19,20]. These variable results warrant further exploration [21]. Additionally, the majority of studies only used a quantitative assessment of effectiveness [12,13,15,16,18,19,20]. The incorporation of qualitative research within these studies could have assisted in the interpretation of the food security outcomes [21].

The Nutrition Education and Skills Training (NEST) program is a food literacy intervention founded in 2014, by OzHarvest, a national Australian not-for-profit food rescue organization. The NEST program is yet to be formally evaluated, hence, the aim of this study was to conduct a descriptive evaluation study into the value of OzHarvest’s NEST program in promoting food security and food literacy, and to identify the barriers and enablers that participants experienced in sustaining food security, and in utilising their food literacy skills beyond the NEST program.

## 2. Materials and Methods

### 2.1. The NEST Program

The NEST program is a 6-week, 15-h guided public health nutrition program, which aims to improve the nutrition, food literacy, and cooking skills of low-socioeconomic Australian adults. Trained NEST facilitators (university qualified nutritionists and dietitians) travel to organisations, such as rehabilitation and health services, food pantries, community centres, and housing support services, with all food, equipment, and educational materials required for the program delivery. Each session integrates a series of nutrition activities, goal setting, and practical cooking lessons, utilising recipes from OzHarvest’s Everyday (photo-based) Cookbook, culminating in the sharing of a meal together. Table 1 provides a detailed outline of session content.

OzHarvest’s nutrition team developed the program to align with the Australian Dietary Guidelines (ADG) [22], and the most recent state/territory-based healthy eating strategies, with support from Deakin University. The program is underpinned by social cognitive theory, as it incorporates a focus on building self-efficacy (belief in one’s ability to successfully perform a behaviour) [23]. Self-efficacy is posited by social cognitive theory as the most important determinant of health behaviour change, affecting how much effort is put into a task and the outcome of that task [23]. The practical demonstrations and regular revision of food selection, preparation and cooking skills were intended to help enhance self-efficacy. It also incorporates a focus on modelling (through skilled facilitators), and interactions between participants and their (food) environments.

NEST incorporates best practice and sustainable public health program evaluation approaches. A process and outcome evaluation framework was implemented to systematically monitor, sustain, and adapt the program, to ensure sustainability, integrity, and fidelity [24]. All NEST facilitators are provided with standardised training and provided with lesson plans outlining the structure, timeframe, and teaching approaches required for successful program delivery. The NEST program manager and trained NEST program auditors conducted observations of the sessions, and debriefing with NEST facilitators and auditors occurred, to ensure fidelity to the intervention plan.

The objectives of the program are to: (1) improve participants’ food literacy, (2) increase consumption of core foods aligned with the ADG, (3) decrease consumption of discretionary foods and drinks, (4) reduce household food insecurity, and (5) increase social engagement (see Supplementary File S4 for a detailed program summary).

### 2.2. Study Design

**Figure 1:**
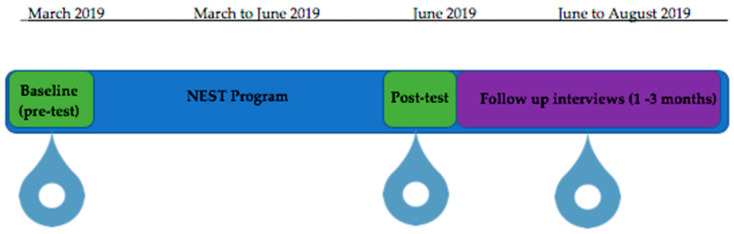
Study design and timeline.

A descriptive evaluation study comprised of pre-post surveys and follow-up interviews with NEST participants was conducted (Figure 1). The pre-post survey data were used to examine the changes in participant’s dietary intake, food security, and food literacy. The interviews provided some explanatory framework to the quantitative results, by providing an in-depth description of participant’s perspectives of their food security and food literacy outcomes and identification of the barriers and enablers to food security that participants experienced beyond the NEST program.

The study was designed to align with the consolidated criteria for reporting qualitative research (COREQ) checklist [25] (see Supplementary File S1). The mixed methods approach provided complementary data for methods’ triangulation, which enabled a more comprehensive examination of the study objectives [26]. The qualitative study was approved by the Deakin University Human Research Ethics Committee (HEAG-H 55_2019) and informed consent was obtained from interviewees. The inclusion of the pre-existing, non-identifiable pre-post survey data received ethics exemption (DUHREC-2019-290).

#### 2.2.1. Pre-Post Surveys

OzHarvest provided pre-existing, de-identified pre-post survey data, with no control group, from a convenience sample of seven NEST programs that were held in Melbourne, Sydney, and Newcastle. The organisations hosting the NEST programs were responsible for the recruitment of participants; the only eligibility criteria provided to agencies was age (18+ years). When organisations sign up to the program, they are provided with a toolkit of posters and flyers to assist in promotion and recruitment of participants that are accessing their services. Each NEST program consisted of groups of 6 to 15 participants. NEST facilitators were trained to distribute the surveys and data was collected from participants before the NEST intervention (baseline), and immediately after the last NEST session (March–June 2019). Fifty-six participants were recruited into the NEST program and completed the pre-survey, and 32 participants completed the post-survey. After removal for attrition (*n* = 24) and incomplete surveys (*n* = 11), the final data set included 21 matched pre-post survey responses.

Due to the lack of validated and reliable survey tools to accurately measure the effect and outcomes of food literacy interventions in low-income population groups [27], a quantitative survey was developed in consultation with key stakeholders. The pre-post survey included 32 core questions, 10 demographic questions (baseline only), and 6 program satisfaction questions (post-intervention only) (see Supplementary File S2). Where appropriate, specific questions that were previously used to assess the impact of other food literacy interventions were incorporated.

##### Food Security

Food security was measured using the 6-item United States Department of Agriculture (USDA) food security measure [28]. This 6-item measure has greater validity and reliability than other single-item food security measures. The 6-item measure has been shown as an effective tool in identifying food insecurity with minimal bias and reasonably high specificity and sensitivity when compared with other measures [28]. However, this food security measure refers to the past 12 months; this would result in overlap of timeframes for the 6-week intervention, hence a shorter timeframe of 6 weeks was substituted. A key limitation of this approach is that validity of items assessing food insecurity over this shorter timeframe is not well-established.

##### Food Literacy

Cooking confidence and self-efficacy were measured using a tool that was adapted from Devine et al. [29] and Barton et al. [30] These items used a 5-point response scale, ranging from “not confident” to “extremely confident”. The self-reported frequency of key food behaviours was measured using a tool that was adapted from the Food Cents program [31] and Wrieden et al. [32]. Knowledge of recommended intake for fruit and vegetables was measured by items validated by Devine et al. [29], and assessed the number of servings of fruits and vegetables the respondent thought people should eat every day for good health. Knowledge on reading nutrition information panels was adapted from Pettigrew et al.’s [33] work.

##### Dietary Intake

Change in daily fruit and vegetable consumption was captured using self-reported questions of fruit and vegetable intake (serves per day), and based on the 1995 National Nutrition Survey [34]. These questions have been previously used in studies with low-income adults in Australia [31,35]. Prompt cards showing one serve were added to aid respondents, consistent with the most recent Australian National Health Survey [36]. Water consumption was measured using a tool from Ball et al.’s [31] study with low-income Australians. It has been adapted from tap water to cover all plain water. The daily intake of sugar-sweetened beverage consumption was based on the National Health Survey [36]; it also included a prompt card showing one serve of various sugar-sweetened beverages. Key discretionary foods—potato crisps or salty snack foods, chocolate or lollies, cake, doughnuts, sweet biscuits, pies, pasties, sausage rolls, fast foods (e.g., McDonalds, KFC), pizza (shop bought or homemade) were measured with a food frequency questionnaire (FFQ). The FFQ did not assess portion sizes, so one serving of the food (based on the ADG) [22] was assumed to represent each eating occasion. These questions have been successfully used with a low-income sample, and were sensitive enough to detect changes in intake resulting from a behavioural intervention [31]. The questions were adapted to fit the timeframe of the NEST study, to ensure the pre and post surveys compared consistent timeframes of 6 weeks.

Participants self-completed, except for those with low literacy (identified by the host organisation staff), to whom the survey was read aloud by trained NEST facilitators and volunteers for a verbal response. The surveys were designed to be completed in approximately 15 min.

#### 2.2.2. Interviews

Qualitative semi-structured interviews were conducted with a sub-set of participants who volunteered to be interviewed, from the same sample of seven NEST programs. All participants (*n* = 56) from this sample of NEST program locations were notified of the opportunity to participate in the interviews, through recruitment posters and flyers that were distributed at the NEST program locations by NEST facilitators or the organisation staff that hosted the program, in April to August 2019. This was considered the most appropriate method of recruitment due to the ‘hard to reach’ nature of this demographic [19,37,38]. No reimbursement or incentive was provided to participants.

Nineteen participants elected to participate in the interviews; two did not met the eligibility criteria of attending at least four of the six program sessions, and so seventeen participants were interviewed by the lead author (EW). The interviews ranged from 13–59 min long (average time 29 min), and due to the contingencies of interstate fieldwork, interviews occurred face-to-face (*n* = 16) or via video call (*n* = 1) [39]. This sample size and approach is in line with other qualitative food security studies published with 15 to 40 interviews [38,40,41]. Interviewees had also participated in the pre-post surveys, but due to the removal of incomplete surveys (*n* = 11) some of the interviewees survey responses may have been removed from the final results.

A semi-structured interview guide (see Supplementary File S3) was developed from the research objectives, the UK Medical Research Centre for Process Evaluation of Complex Interventions guidelines [42], and the NEST Program Evaluation Framework Recommendation Report [43]. The interview guide enabled investigation into the impact of NEST from the participant’s perspectives. Questions were developed about participant’s food utilisation, cooking confidence, food literacy skills and behaviours, and food environment. Interview questions also permitted exploration into the barriers and enablers that participants experienced, during or post intervention. Interviewees were also asked to provide demographic information, including the 6-item USDA food security measure post-interview, to provide the background characteristics and food security level of interviewees.

### 2.3. Analysis

#### 2.3.1. Pre-Post Surveys

Statistical analyses were performed on survey data using STATA statistical software release IC 15.1 (StataCorp, LLC, USA) [44]. Descriptive statistics were obtained by calculating the mean and standard deviation (SD) for continuous variables (food behaviours, cooking confidence, and dietary intake behaviours), and proportions for categorical variables (demographics). The dichotomous variables for the nutrition knowledge questions (correct or incorrect) were assigned values to convert them to a score of 0 or 1. Incorrect responses were assigned a score of ‘0’ and correct responses were assigned a score of ‘1’; the sum of all nutrition knowledge items were calculated as a single continuous measure (maximum score of 5), and the mean and SD of this summary score was calculated. For the 6-item USDA food security measure, proportions of each categorised response were obtained. Additionally, following the USDA food security protocol, affirmative responses ‘often true’ and ‘sometimes true’ were assigned a score of ‘1’. Negative responses ‘never true’ or ‘don’t know’ were assigned a score of ‘0’ [28]. The sum of affirmatives to each response indicates an individual’s level of food security. Once the food security levels were calculated, this was treated as a continuous variable. The Shapiro–Wilk W test was used to check for normality. Due to the sample size and lack of normal distribution across the data sets, the nonparametric Wilcoxon signed-rank test was used to compare continuous variables at pre and post intervention (*p* < 0.05 for all tests). Power calculations were not conducted, as this was an exploratory study.

#### 2.3.2. Interviews

After interview completion, the audio files were transcribed verbatim by the lead author (*n* = 3) or a transcription service (*n* = 14). A general inductive approach for the analysis of the qualitative part of this study was undertaken. The COREQ checklist was followed to strengthen analysis and interpretation conformability [25]. Transcripts were analysed thematically to provide a rich and detailed account of the data [45]. Analysis involved actively identifying, coding, and classifying patterns and themes related to the study objectives. Authors (EW, RL) met to discuss emergent themes and coding processes. The research questions and the four dimensions of food security formed the initial framework for coding. The significance of emergent patterns were then interpreted and theorised, to explore their broader meanings and implications [46]. Themes and subthemes were then created through amalgamation and separation of these initial codes. The data were managed using computer assisted software (NVIVO 12 Plus), which increased the efficiency and constancy of the results [45]. Descriptive statistics were conducted on the interviewees’ demographic data using Microsoft Excel. All participants were provided with a summary of the results at the conclusion of the study.

## 3. Results

### 3.1. Pre-Post NEST Survey Results

#### 3.1.1. Demographics

Table 2 provides a summary of the twenty-one participants that completed the pre-post surveys; the majority were female (57.1%), unemployed (81%), and almost all prepared at least some of their weekly meals (90.6%). Over half were residents of assisted living or short-term emergency care facilities (52.4%). OzHarvest was providing rescued food to three of the seven organisations included in this study. 

#### 3.1.2. Food Security

At baseline, 61.9% of participants were classified as food insecure, as low food security (score of 2–4: 47.6%), or very low food security (score 5–6: 14.3%) (Table 3). At post-intervention, 42.9% of participants were classified as food insecure, having low food security (score of 2–4: 33.4%) or very low food security (score 5–6: 9.5%). The mean food security score significantly decreased between baseline (pre) and post-intervention (pre vs. post food security score mean ± SD: 2.33 ± 1.74 vs 1.67 ± 1.71, *p* = 0.030), suggesting an improvement in participants’ food security, from baseline to post intervention.

#### 3.1.3. Cooking Confidence, Food Behaviours, and Nutrition Knowledge

Table 4 shows a statistically significant increase between baseline and post-intervention in the average cooking confidence score (range 1–5) (*p* = 0.001). All individual cooking confidence measures improved, except confidence in one’s ability to buy healthy food on a budget. The combined average food behaviour score showed a significant increase in health-promoting behaviours from baseline to post-intervention (*p* = 0.006) (Table 4). Most food behaviour scores were significant, except for reading the ingredient list, looking at the price per kilo when shopping, changing recipes to make them healthier, and adding salt to food when cooking. A significant increase in the average total nutrition knowledge scores from baseline to post-intervention was found (*p* = 0.033) (Table 4). 

#### 3.1.4. Dietary Intake

Average daily vegetable intake increased significantly from baseline to post-intervention by more than half a serve (*p* = 0.043) (Table 5). There was a significant reduction in the consumption of sugar-sweetened beverages by almost half a serve (*p* = 0.017). The overall average consumption of discretionary foods decreased by almost half a serve, although this difference was not statistically significant (Table 5).

### 3.2. Interview Results

The interview findings provided a deeper understanding of the pre-post survey results from the NEST participant’s perspectives. Seventeen interviews were conducted with past NEST participants, within two to ten weeks of program completion. The majority were male (64.7%), born in Australia (70.6%), living in social housing or rehabilitation centres (58.8%), with a household income of <$AUD 575 per week (58.8%) (Table 6). Their ages ranged from 24 to 80 years, with a mean age of 48.8 (±16.4) years. Overall, 47% of interview participants were classified as food insecure, with 23% experiencing very low food security. Nine themes were identified to provide insight into the impact, barriers, and enablers that participants experienced in sustaining food security and utilising their food literacy skills beyond the NEST program. These were then classified and presented as three major themes: (1) NEST improves food literacy and food utilisation; (2) enablers for food security; (3) barriers to food security.

#### 3.2.1. NEST Improves Food Literacy and Food Utilisation

The food literacy skills participants gained from the NEST program were reported to help achieve the food utilisation dimension of food security. The NEST participants described improvements to food literacy in terms of cooking confidence, food preparation and cooking methods, food selection and eating behaviours, and stretching food budgets and saving money.

##### Cooking Confidence

Participants described confidence in being able to shop for food, read and follow recipes, and prepare foods safely. Participant 5 explains, “the cooking experience… this was the first time… and you don’t realise how good you are until you have a go at it and I thought, there you go that proves that I can do something when I want to”. Participants described that this confidence brought feelings of pride and self-efficacy. According to Participant 14, “pride’s a big thing in life… I did this and the fact that I did it, I was proud of myself”. This new confidence in cooking motivated participants to utilise their new skills by increasing their cooking frequency. Increased cooking confidence also led some participants to experiment by cooking with new foods and recipes. Participant 14 shared, “I’ve been cooking things since I’ve done the program, which I wouldn’t have done before, like pasta and the way I prepare my vegie’s… I enjoy that, and I experiment with that… I did one yesterday, which was the nicest I’ve ever got it”. This improved confidence led some participants to share future plans to utilise these skills beyond their current circumstances, with plans to cook for their families once they were out of rehabilitation, or to cook for themselves once they left their assisted living residence (where food was typically provided to inhabitants and self-catering was limited).

##### Food Preparation and Cooking Methods

A shift to prepare meals using healthier ingredients and cooking methods was evident in the perceived outcomes since the NEST program. As described by Participant 10:

From the fifteen months I had been in <rehabilitation> we used to have four or five days a week of fried foods… Mainly from awareness, mainly from affordability… So, what the NEST program was teaching us… we have been incorporating that into our weekly menus… a healthier way of cooking with healthier oils… to do chips steamed or oven… we don’t need to put everything in the deep fryer, which is what we were doing.

Many participants shared that they were incorporating more vegetables, baking instead of frying, and choosing fresher products, rather than packaged or pre-prepared meals. Interviewees also described learning how to substitute more expensive ingredients for cheaper ones, to extend meals. Replacing meat or extending it by adding other protein sources (such as legumes) was a commonly reported strategy. Participant 3 described, “it’s sort of helped to eat—like before the program I wouldn’t know if you can replace this with this… You can have legumes or soybeans or something like that instead of meat”. Another strategy was substituting more expensive cuts of meat for cheaper ones, as Participant 16 explained, “I bought a chook but I roasted it myself and I’ve been using the roast chicken from that for my lunches during the week... Like instead of just buying…chicken fillets and cook them… A whole chook cost me like $4 or $5, where the chicken fillets are like $4 or $5 for three… just making slightly smarter options”. Substituting ingredients was a particularly important food literacy skill for participants that were receiving rescued food, as participant 12 (from an agency that receives a weekly food delivery) shared, “I <was> made aware of what you can substitute things for… what you can add in, what-you can change things for other things. As long as it’s similar… I look at squash being zucchini, they’re just a different shape, different colour but essentially…do the same thing”. The key benefit of substituting and replacing ingredients was that participants were able to more effectively utilise their ingredients to “make a big meal and have it stretched over a couple of nights or lunch and dinner”.

##### Food Selection and Eating Behaviours

Participants described that the NEST program increased their understanding of healthy eating practices and as a result were more mindful when selecting foods to eat. As participant 1 explains, “It gave me some insight into healthy eating, what are good options and what I should be eating…we got information on that thing about the salts and the sugars and all that … and how much is appropriate fat…and which ones are not so good and all that”. They continued to explain that when they buy food, they “look at those items and you say which ones are appropriate and which ones are not for your own overall health”. Several other participants reported that they “learnt about the reading on the packets” and “check the labels a bit more carefully now”. Increased variety “in different types of food” and “balance in cooking” were also reported, with participants explaining that they’ve swapped to wholemeal grains and cereals and choosing low fat varieties of dairy products.

##### Stretching Food Budgets and Saving Money

Several participants explained that in the NEST program, they learnt about affordable food options, food storage techniques, and shopping strategies (e.g., looking at price per kilo, using a shopping list etc.), which assisted them to make their food budget stretch further. Acquiring food budget skills was particularly important for Participant 16:

When I became unemployed it was like we can’t have those easy quick meals because they’re not cheap. So, I had to re-learn myself how to cook meals again and make them stretch… I just got so caught up in easy buy stuff, and like just for the convenience of it all. Because we went from having two fairly good wages to one wage that barely covered anything, we really had to learn how to–just the basics again. I know it sounds so silly, but it’s true…

A key part of stretching budgets for interviewees was linked to food literacy skills of storing and utilising foods that participants already had, as Participant 11 described that NEST taught them, “How to make use what you have in the cupboard, which saved us a lot of money in the long run, and gave us healthier food… our money budget for shopping has reduced, and we are coming back with change as well, we used to run out”.

#### 3.2.2. Enablers for Food Security

Some participants experienced enablers that supported them to remain food secure (or less at risk of very low food security) beyond the NEST program. The enablers that participants reported were: (1) receiving and providing support to family or friends and (2) provision of charitable food.

##### Receiving and Providing Support to Family or Friends

Participants described that regular support from family or friends was an enabler for sustaining food security or protecting from the worst aspects of food insecurity. Some participants shared that they had a family member or friend provide regular support through the provision of money, groceries, and/or cooked meals. For others, support was provided through disseminating the knowledge and skills gained in the NEST program. For Participant 16, this meant sharing the information she learnt from the program with her husband. She explained that her husband, “Is happy to go shopping for me sometimes too now which is amazing. He hates any type of shopping. So, to see him go grocery shopping is a big thing because… he knows now to look at the bargains, I’ve taught him how to do that as well”. For others, the knowledge and skills gained from the program were sustained after the program, because participants were motivated to support their family and friends. As Participant 13 shares: “I will stay on that path, you know, because it’s better. And I’ve got kids to play with… I found it very helpful to get some ideas… I think about when I come home, how will I cook and how healthy I can eat… I want to cook for my wife, my kids more healthy… and learn more about healthy living”.

##### Provision of Charitable Food

Most participants reported accessing some form of charitable organisation for food, but their reliance on it varied. Some interviewees obtained almost all of their food from food charities; most were accessing a food charity once a week. Meanwhile, for participant 16, food relief was accessed regularly, but only for a short-period of time; she shared, “So, up until recently I was unemployed… I was going to <organisation name> for food donations. Like a week under five months I was unemployed for. So, I was going to <organisation name> for all that time to help…. I felt that <organisation name> and OzHarvest were all there to help me and my husband get ourselves back on our feet and get us through this really bad, dark, <expletive> situation”. Interviewees were living in a rehabilitation centre that provided all ingredients and a meal roster, for all residents to take turns preparing meals. In these instances, the provision of food extended budgets and helped to save money, so that the organisation could “spend the money on other things that are necessary around the house, which is good. So OzHarvest does save us a lot of money around the place”.

#### 3.2.3. Barriers to Food Security 

Participants still faced key barriers to food security, these were described as: (1) lack of economic access to food, (2) pre-existing health issues, and (3) provision of charitable food.

##### Lack of Economic Access to Food

Access to food includes economic access or affordability of food (17). It is evident from the themes above that the NEST program supported participants to stretch food budgets and save money, but the difficulty of not having enough income to afford food remained. Participant 8, for example, explained

Unfortunately, due to the cost of living and expenses, making food and getting food is quite hard… you need a roof over your head and you need to be able to pay bills and stuff, so try and pay them off... Then food generally comes last. It just means lack of food a lot of the time… there’s no point in me saying, ‘Yes, I’ll cook three meals a day and I’ll make sure to include every type of food.’ Because of course, that would straight away be a fail because it’s not accessible.

Many participants struggled to afford safe, nutritious, adequate, and culturally appropriate food. For many, this meant forgoing fresh fruit and vegetables. Participant 7 explained, lack of money meant that she “can’t really get the veggies you need in your diet, you can’t get a lot of the fruit you need”. As a result, she turned to cheaper foods, “that will fill you up, keep you energised for ages, but not break the ability to pay the bills”. She described that this “has been upsetting” as she and others want to eat healthier, but “it’s all about living with what you can”.

##### Pre-Existing Health Issues

Mental health issues were also reported by some participants as a barrier to food security. Participant 1 explains, “some barriers like to go out and shop and get my own healthy food. Sometimes the barrier’s a bit of anxiety… worried about people looking at me and all that”. Pre-existing mental health issues were found to not only impact the participant’s ability to physically access the food, it was also mentioned as a barrier to utilising the skills developed from NEST. Participant 8 explained that she and other participants “were facing barriers in regard to ability to get the food or have energy to cook”. This was echoed by Participant 2, who shared, “I haven’t been cooking much lately because I’ve been a bit depressed, but yeah, normally on Saturday’s I’ll cook a roast or something for me and <name of friend>”. Drug and alcohol addictions were also identified by participants as obstacles to them becoming both food secure and healthy. Participant 10 described that he “was a lot fitter when I was younger and I lost that due to my drug addiction, yeah umm I’ve lost a lot of sort of wants. The lifestyle I was living was nearly impossible to be doing healthy living you know”.

##### Provision of Charitable Food

As discussed above, most participants accessed food from charitable organisations, and some viewed this as an enabler for food security. However, for others, the types of food provided by charitable organisations were considered a barrier, as they were not necessarily nutritionally adequate. Participant 1 explained, “I haven’t really been cooking for myself that much. I haven’t changed that. Mainly we get meals here…. The meals here are not too bad, health wise. The desserts can be a barrier because we have dessert as well”. Participant 10 explained that, while NEST encouraged their household to “want change and want healthy eating, a lot of goodies come on the OzHarvest truck”. It is evident that the key nutritional messages from the NEST program were at odds with some of the rescued food provided by OzHarvest and other organisations. This was also reported by Participant 8, who shared:

we’re given a bag of food and sometimes it’s quite simply a loaf of bread, a little bag of cereal, and some muesli bars and maybe one packet of pasta. Which means it doesn’t go far… as grateful as we are for the amount of food we get, it’s not possible to make certain recipes out of it. And quite simply put, if there’s no ability to cook… it’s just going to actually make you feel more upset.

## 4. Discussion

This descriptive evaluation study investigated the NEST Program’s impact on food security and food literacy, and identified key barriers and enablers that participants experienced in sustaining food security and utilising their food literacy skills beyond the program. Both interview and survey findings suggest that NEST had a significant effect on food security and food literacy. The pre-post survey data suggested an improvement in participants’ food security from baseline to post intervention. and improvements in food literacy (cooking confidence, food behaviours, nutrition knowledge), and some measures of dietary intake (vegetable consumption, sugar-sweetened beverages and salty discretionary foods).

The qualitative interview data analysis yielded themes that described how the NEST program changed participants’ food literacy and food insecurity, as well as the barriers and enablers to food security that participants experienced. The program was shown to improve food security and increase food literacy skills, enabling participants to stretch their food budgets, select and cook healthier foods, and save money. Support from family or friends and the provision of food from charitable organisations enabled some participants to feel more food secure beyond the program. Participants still faced key barriers that were linked to other dimensions of food security, including lack of economic access to food, and pre-existing health issues. Interestingly, the provision of charitable food was also considered a barrier to some participants, due to the lack of variety and nutritionally adequate foods, which hindered participants in utilising the skills and knowledge they reported obtaining from the NEST program.

### 4.1. The Role of NEST on Food Security and Food Literacy

Food security improved by 28% from baseline to post-intervention among participants. This is slightly higher than that reported in other similar interventions, which have found a 12.9% to 25% improvement in food security [12,13,15], or no improvement [18,19,20]. Participants described that the skills they gained from NEST specifically assisted them to stretch food budgets and save money. These findings are supported by previous research on food literacy interventions, which found change in attitudes and improved ability to stretch food dollars [12,13,14,15,18,20,47,48,49].

The NEST program is underpinned by social cognitive theory, and the program aims to build self-efficacy in its participants. Increased self-efficacy has been shown to improve an individual’s capability across both the access and utilisation dimensions of food security [8,12]. However, participants’ confidence in ability to buy healthy food on a budget showed no significant change from pre- to post-program. This may be due to the fact that a number of participants were living in housing where at least some of their food was provided. Alternatively, while gains in skills may help improve confidence to buy healthy food on a budget, this may be limited in cases of a particularly low budget, such as those on unemployment benefits [50]. This experience was demonstrated in the qualitative results; interviewees reported their desire to shop and prepare healthy meals, but were restricted by very low income/unemployment benefits. Meanwhile other interviewees reported improvements in self-efficacy and confidence to eat healthy on a budget, to the extent that participants reported the new skills and greater confidence they gained from the program, and their sense of pride and motivation to utilise their newly developed skills. Begley et al. [8] highlight the importance of ensuring that evaluations of food literacy interventions are sensitive and comprehensive enough to capture emerging food literacy behaviours of individuals experiencing food insecurity. A mixed methods approach was valuable for examining the impact that NEST had on food security, because it was able to capture the participant’s emerging self-efficacy and food literacy skills [8].

### 4.2. Barriers and Enablers to Food Security

The results of the NEST program on food insecurity are promising, however, understanding the enablers and barriers to food security for these participants is essential to properly consider the role interventions like NEST play in promoting food security. There is evidence that the provision of food assistance together with a food literacy intervention improves food security [8,18]. This was confirmed by the qualitative component of this study, with some participants acknowledging that attending the program, together with receiving charitable food, was beneficial for them. Other food literacy programs, such as the US program Supplemental Nutrition Assistance Program, provide education sessions together with access to specific nutritious foods, rather than variable charitable food, which have been positively reported to improve food security outcomes [51]. However, it is difficult to determine what outcomes in food security status can be attributed to the food literacy intervention, and what can be attributed to the food provision component.

Unlike the US, Australia has no national system of welfare food provision based on income, rather, charitable food is available, but can vary in quantity and quality. Acquiring food from charitable organisations in this way is widely considered as a socially unacceptable way of procuring food [52,53,54,55]. Hence, as Meiklejohn et al. [38] highlighted, food literacy intervention participants that are receiving food assistance cannot be reported as food secure. This was apparent in the present study, as some participants were classified as food secure at both time points using quantitative assessments, even though the qualitative interviews revealed that most NEST participants were accessing charities for food. The validated tool used to measure food security in this study is the standardised 6-item USDA food security measure [28]. This tool has been critiqued, because it only assesses one of the four dimensions of food security; economic access [56]. As such, this tool does not measure whether participants are accessing charities for food, nor the frequency of this access. The findings from this study further confirm the need for a measure that reflects all dimensions of food security [57,58,59].

Both quantitative and qualitative data demonstrated that NEST improved the utilisation dimension of food security. However, the qualitative data revealed that lack of economic access due to the price and affordability of foods remained a key barrier to food security. This is not surprising, as most interviewees (58.8%) reported very low income levels, relying on ≤AUD $575 per week, compared to the average weekly wage of approximately AUD $1250 in 2019 in Australia [60]. Recent studies have estimated that, for a low-income household to meet the Australian Dietary Guidelines, they would need to spend 30% to 70% of their budget on fruits, vegetables, and healthy food items [9,61,62]. Although NEST promotes strategies to eat healthy on a budget, the analysis of the interview data found that the participants still struggled to afford nutritious, adequate, and culturally appropriate food after the program. Participants reported wanting to purchase fruit and vegetables, but they were perceived as too expensive, so “cheaper” and “more filling” energy-dense foods were selected. This approach has been repeatedly recognised as a practice that low-income Australian households have utilised to feed families [8,63]. These findings highlight that food literacy interventions play a role in improving food security, but cannot address food insecurity alone [8,9].

### 4.3. Implications

#### 4.3.1. Implications for Research

This study adds to the body of literature around food literacy interventions, which to date, has shown a varying effectiveness of impact on food security [12,13,18,20]. The barriers and enablers to food security found in this study highlight limitations in the 6-item food security measurement tool, specifically, its inability to measure access and frequency of receiving charitable food. This tool was also designed to measure a 12-month timeframe, so its ability to measure changes in this 6-week program timeframe are questionable. Future research on the effect of food literacy interventions on food security should adopt a mixed-methods approach, with a validated food security tool that can measure all four dimensions of food security. A comprehensive measure, such as the measure currently being developed by Kleve et al. [58], should be considered, to provide a more rigorous and reliable way to examine the role that food literacy interventions play on all dimensions of food security.

Further research is also required, that investigates and addresses the food environment and external factors related to food security. For example, programs should ensure that food literacy intervention participants can access affordable high-quality foods in the regions where interventions are conducted [17]. As food insecurity can be chronic or transitory [60], such work should also examine whether food security improvements can be maintained over time. This is particularly important, as the strength of food literacy interventions against challenging circumstances such as job loss, addition of members to the household, and illness is unknown. Qualitative and/or mixed research methods are recommended to explore these external factors, and other dimensions of food security for food literacy intervention participants.

#### 4.3.2. Implications for Policy

Food insecurity occurs at a global, national, community, household, and individual level, and is the result of multiple and complex environmental and individual factors [17,64]. Environmental factors may include food access and supply, government food policies and taxes, living costs, welfare support systems, and affordability of food [7,65]. Individual-level factors include access to employment, income, nutrition knowledge, and cooking skills [8,63]. However, the most consistently reported underlying determinants of food insecurity are the cost of food and household income [66,67].

This enquiry into the NEST program suggested an improvement in participant’s food security from baseline to post intervention. However, education alone cannot change food prices or address other upstream determinants of food security. The barriers to food security that participants faced were related to other dimensions of food security, indicating the need for multiple approaches to food insecurity, including immediate food assistance, food literacy interventions, and structural level initiatives. Economic access to nutritious food was highlighted as a key barrier to food security, hence the core part of this structural level initiative would ensure that all citizens have a ‘right to food’, achieved through the provision of an adequate living wage [8]. This study provides further evidence on the need for structural-level initiatives, such as increased government assistance, higher welfare payments (particularly Newstart in Australia), and regulations that create healthier and more affordable food environments for all [8,9,60].

#### 4.3.3. Implications for Practice

This study found that a theoretically grounded program that caters to the needs of low-income groups can play at least a short-term role in building skills and addressing food literacy and food security. Therefore, such programs could comprise a part of broader community-based approaches to addressing food insecurity. However, aspects of the program could be improved. For example, the key nutritional messages from the NEST program were at odds with some of the rescued food provided by OzHarvest and other organisations. There is a need for charitable food organisations to develop nutrition guidelines, to ensure that they are providing a healthy food environment for their participants.

The qualitative findings found that mental health issues were barriers to food security. There is an increasing amount of research exploring the bidirectional impact of mental health issues and nutrition [68]. Cooking workshops conducted with people with serious mental illness have been shown to be an empowering and positive experience, with participants reporting improved social engagement and healthier food choices [69]. This provides an opportunity to incorporate positive mental health messages within food literacy interventions. For example, a module on nutrition and mental wellbeing is under development, to be incorporated into the NEST program. Increased self-efficacy has been shown to improve individual’s capabilities across both the access and utilisation dimensions of food security [69]. However, this finding highlights the need for further exploration to develop strategies to support individuals experiencing mental health issues and food insecurity in the community.

Support from friends and family was reported as an enabler for sustaining food security or protecting from the worst aspects of food insecurity. There is sound evidence that engagement from social support can increase the effectiveness of dietary interventions [70]. However, several participants shared that the knowledge and skills gained from the program were sustained beyond the program because the participants were motivated to support their family and friends. This is a noteworthy motivation, and suggests that goal setting components of food literacy interventions may be more successful if goal setting does not solely focus on the individual participant’s behaviour change, but encourages participants to set goals for sharing this knowledge with others. This approach has now been incorporated into the NEST program, where, as part of the goal setting activity we encourage participants to reflect on who they could share this information with.

The pre-post surveys reported insignificant changes in participant’s confidence to cook healthy on a budget, whilst the qualitative results showed that tips on stretching food budgets were beneficial to participants. This provided an opportunity to review the way NEST facilitators were delivering the content and amendments to the NEST facilitator handbook were made to ensure useful advice on budget healthy eating was explicitly incorporated into each module. For example, highlighting that adding lentils to spaghetti bolognaise not only provides additional protein and fibre, it also can save money, as 500 g of beef mince costs AUD$8.00, whilst a 475 g tin of lentils costs AUD$2.00.

Overall, these findings support the current public health research that food literacy interventions can play a small, but important role in the adoption of healthier food behaviours [70].

### 4.4. Strengths and Limitations

This descriptive evaluation study was strengthened by its mixed methods approach, through the use of qualitative data that provided some explanatory framework to the quantitative results. The first author is an employee of OzHarvest in the role of National NEST Program Manager, and was involved in the development and delivery of the NEST program. The consequent familiarity with the program and experience working with people experiencing food insecurity is often seen as a strength in qualitative research [38,71]. This experience enabled the researcher to build rapport and trust quickly with participants, for rich data collection [37]. This role may, however, have also introduced researcher bias, with findings likely to reflect aspects of the first author’s experience and knowledge, that were difficult to remove from their analytical lens [71]. The qualitative research (COREQ) checklist [25] was followed, and reflexivity was used throughout data collection and analysis to minimise this bias [71].

A limitation of this study is the relatively small sample size, and the lack of control or comparison group. The strongest level of evidence of a food literacy intervention’s impact is generated from randomised control trials that include a control group [72]. However, this was not feasible within OzHarvest’s resources and the timeframe of this research project, hence, assessment using a pre-post test design with no control group was conducted. This approach has been used in other food literacy evaluations [72], and is stronger than a post-test only design, which offers very weak evidence of change [72]. Other limitations include the high attrition, and reliance on self-reported data, which lacks the validity of objective measurement tools [73]. Additionally, social desirability bias is possible in both the pre-post survey and interview data, as participants may have reported greater changes in food literacy and better food security than they actually experienced. Finally, the interview eligibility criteria required participants to have completed at least four of the six NEST sessions. Interviewees may have experienced more stable lives (more food secure), and therefore had greater ability to respond to the program. Hence, there are limitations to the generalisability of the results. Despite these limitations, conducting randomised control trials in real world environments is challenging and more studies using quasi-experimental designs are required [72]. Self-reported surveys continue to be the primary tool used in food literacy intervention evaluation, due to their low cost, minimal participant burden, and ease of administration [74].

## 5. Conclusions

This descriptive evaluation study explored the value of OzHarvest’s NEST program in improving participants’ food security and food literacy. The results of this study indicate that the NEST program may improve food security and food literacy. The program was shown to increase food literacy skills, enabling participants to stretch their food budgets, select and cook healthier foods, and save money. Despite the positive findings of this study, the obstacles to food security reported by participants confirm that food literacy interventions alone cannot impact the other determinants of food insecurity [8]. NEST provided food insecure individuals with food literacy skills, enabling them to make meaningful changes to their food utilisation (a key dimension of food security). Food literacy interventions may have a role to play in addressing food insecurity as part of a multi-faceted approach. This multi-faceted approach should include immediate food assistance, food literacy interventions, and structural-level initiatives, to tackle this social and health issue that impacts more than one million Australian households [6,66].

## Figures and Tables

**Table 1:** NEST (Nutrition Education and Skills Training) Program Session Details.

*Session*	Lesson Outline	Teaching Approaches
*Module 1:* *Eat for variety*	Pre-program evaluation survey distributed to participants. Introduce facilitator, OzHarvest, NEST purpose, scope, structure. Build rapport with participants and explore existing knowledge and experiences with cooking and healthy eating. Present and discuss the Australian Guide to healthy eating, eating a variety of foods from 5 food groups, increasing fruit and vegetable consumption. Interactive practical activity: The Healthy Plate Model or Serves and Food Group Activity. Facilitate goal setting that enables participants to develop a SMART goal that is suitable for their circumstance. Practical cooking activity: Food safety brief, group cooking experience preparing 1–2 healthy recipes. Sharing of meal prepared: Social time, summary of key learnings, choice of next session’s recipes, informal evaluation of the session.	Module 1 is focused on a simple introduction to healthy eating and how to increase fruit and vegetable consumption on a tight budget. Key Learning Area: How to increase fruit and vegetable consumption. Building rapport is essential to ensure participants feel welcomed and included. Icebreakers, interactive discussions and activities are used to assess participant’s nutrition knowledge, eating habits, and cooking skills to tailor the program to participants needs (e.g., choosing a suitable activity) Practical advice is provided in a way that supports the positive features of the participant’s diet, while drawing attention to areas of improvement without being judgemental or discouraging Educational and cooking activities and approaches are applied using key concepts of social cognitive theory. Encourage small group discussion using prompt questions and activities–monitor small group discussion and return full group for overview and confirmation of understanding
*Module 2:* *Eat for Wellbeing*	Welcome to NEST session 2: Recap of previous session with interactive trivia questions and initial group review of goals from previous week. Present and discuss key nutrients and healthier options within the 5 food groups, portion size vs. serve size. Interactive practical activity: My Five Food Groups Plan–Simple or Advanced version Review goals from previous week and refine the previous goal or set a new goal if appropriate. Practical cooking activity: Recap food safety brief, group cooking experience preparing 1–2 healthy recipes. Sharing of meal prepared: Social time, summary of key learnings, choice of next session’s recipes, informal evaluation of the session.	Module 2 continues the introduction into healthy eating but focused on how to increase variety within the core food groups on a tight budget. Key Learning Area: How to increase variety within the core food groups. Ensure participants continue to feel welcome and included. Make sure that the participants don’t feel overwhelmed during the sessions. Remain aware of the participants’ verbal and non-verbal cues when leading discussion and setting goals. Support participants’ in focusing on healthy positive behaviour changes rather than dwelling on unhealthy food choices. Focus on strategies for consuming healthier nutrient-dense foods. Practical advice is provided in a way that supports the positive features of the participant’s diet, while drawing attention to areas of improvement without being judgemental or discouraging. Educational and cooking activities and approaches are applied using key concepts of social cognitive theory. Encourage small group discussion using prompt questions and activities–monitor small group discussion and return full group for overview and confirmation of understanding
*Module 3:* *Eat for Balance*	Welcome to NEST session 3: Recap of previous session with interactive trivia questions and initial group review of goals from previous week. Present and discuss the role of ‘extra’ foods in a balanced diet, label reading, identifying foods high in sugar, salt, and fats. Interactive practical activity: Guess What, Swapping and Switching, Reading Nutrition Information Panels. Review goals from previous week and refine the previous goal or set a new goal if appropriate. Practical cooking activity: Recap food safety brief, group cooking experience preparing 1–2 healthy recipes. Sharing of meal prepared: Social time, summary of key learnings, choice of next session’s recipes, informal evaluation of the session.	Module 3 provides simple ways to identify and swap foods to reduce intake of energy dense and nutrient poor food and drinks. Key Learning Area: How to swap foods to reduce intake of energy dense and nutrient poor foods and drinks. Ensure participants continue to feel welcome and included. Make sure that the participants’ don’t feel overwhelmed during the sessions. Remain aware of the participants’ verbal and non-verbal cues when leading discussion and setting goals. Support participants’ in focusing on healthy positive behaviour changes. Focus on swapping strategies for consuming healthier nutrient-dense foods. Practical advice is provided in a way that supports the positive features of the participant’s diet, while drawing attention to areas of improvement without being judgemental or discouraging. Educational and cooking activities and approaches are applied using key concepts of social cognitive theory. Encourage small group discussion using prompt questions and activities – monitor small group discussion and return full group for overview and confirmation of understanding.
*Module 4:* *Eat for the Environment*	Welcome to NEST session 4: Recap of previous session with interactive trivia questions and initial group review of goals from previous week. Present and discuss the tips to fight food waste, save money, and safe food handling and storage methods. Interactive practical activity: Lasting Leftovers and Store it Safely. Review goals from previous week and refine the previous goal or set a new goal if appropriate. Practical cooking activity: Recap food safety brief, group cooking experience preparing 1–2 healthy recipes. Sharing of meal prepared: Social time, summary of key learnings, choice of next session’s recipes, informal evaluation of the session.	Module 4 provides additional food literacy skills of safe food handling and storage methods and food utilisation strategies to reduce household food waste. Key Learning Area: How to make the most of healthy food on a budget by preparing and storing it safely. Ensure participants continue to feel welcome and included. Make sure that the participants’ don’t feel overwhelmed during the sessions. Remain aware of the participants’ verbal and non-verbal cues when leading discussion and setting goals. Support participants’ in focusing on healthy positive behaviour changes. Focus on swapping strategies for consuming healthier nutrient-dense foods. Practical advice is provided in a way that supports the positive features of the participant’s diet, while drawing attention to areas of improvement without being judgemental or discouraging. Educational and cooking activities and approaches are applied using key concepts of social cognitive theory. Encourage small group discussion using prompt questions and activities – monitor small group discussion and return full group for overview and confirmation of understanding
*Module 5:* *Eat for Choic*e	The charitable agency staff and NEST participants choose from the following modules: **Module 5a: Eating Healthy when Eating Out:** - Dining out tips and traps - Energy dense nutrient poor foods - Healthier swaps **Module 5b: Eating for Infants and Young Children:** - Introducing solids and first foods - Texture progression - Parent-child division of responsibility during mealtimes **Module 5c: Eat to Move (Physical Activity):** - Importance of physical activity - Australian guidelines - Active swaps **Module 5d: Eating for Pregnancy and Lactation:** - Healthy eating guidelines for pregnancy and lactation - Supplement use **Module 5e: Eating for Diabetes Management:** - Food as carbohydrates, protein and fats - Type, amount, and frequency of carbohydrates **Module 5f: Eat from the Supermarket:** - Mock supermarket tour - Health claims and key nutrients - Healthier options in different sections of the supermarket	The Module 5 options provide additional food literacy and nutrition education relevant to the module’s theme. Charitable agency staff and participants choose or vote on the module that is most suitable. Ensure participants continue to feel welcome and included. Make sure that the participants don’t feel overwhelmed during the sessions. Remain aware of the participants’ verbal and non-verbal cues when leading discussion and setting goals. Support participants’ in focusing on healthy positive behaviour changes. Focus on swapping strategies for consuming healthier nutrient-dense foods. Practical advice is provided in a way that supports the positive features of the participant’s diet, while drawing attention to areas of improvement without being judgemental or discouraging. Educational and cooking activities and approaches are applied using key concepts of social cognitive theory. Encourage small group discussion using prompt questions and activities–monitor small group discussion and return full group for overview and confirmation of understanding
*Module 6:* *Eat for Life*	Welcome to NEST session 6: Recap of previous session with interactive trivia questions and initial group review of goals from previous week. Overview of program exploring and affirming healthy eating behaviours achieved from program. Interactive discussion on healthy eating on a budget-comparing prices of healthy vs. less healthy foods, strategies for healthy eating on a budget. Interactive practical activity: Food Cents-Simple or Advanced option Review goals from previous week and refine the previous goal or set a future beyond the program goal. Practical cooking activity: Recap food safety brief, group cooking experience preparing 1–2 healthy recipes. Sharing of meal prepared: Social time, summary of key learnings Celebration of program completion—NEST certificate of completion and participant tool kit (with NEST Cookbook and key educational resources) provided to each participant. Post-program evaluation survey distributed to participants.	Module 5 brings the entire program together and provides strategies for healthy eating on a budget beyond the program. Key Learning Area: How to reduce barriers for long-term healthy eating. Ensure participants feel affirmed in their participation throughout the program. Make sure that the participants don’t feel overwhelmed during the sessions. Remain aware of the participants’ verbal and non-verbal cues when leading discussion and setting goals. Support participants’ in focusing on healthy positive behaviour changes. Practical advice is provided in a way that supports the positive features of the participant’s diet, while drawing attention to areas of improvement without being judgemental or discouraging. Educational and cooking activities and approaches are applied using key concepts of social cognitive theory. Encourage small group discussion using prompt questions and activities–monitor small group discussion and return full group for overview and confirmation of understanding Reinforce and celebrate positive behavioural changes that have occurred across the program and motivate participants to continue with their goals long-term.

**Table 2:** Demographic characteristics of NEST pre-post survey participants (*n* = 21).

Category	*n* (%)
**Gender**	
Male	9 (42.9)
Female	12 (57.1)
**Age**	
18–34 years	6 (28.6)
35–54 years	10 (47.6)
55–74 years	5 (23.8)
**Employment Status**	
Employed	3 (14.3)
Employed—unpaid	1 (4.7)
Unemployed	17 (81.0)
**Education Level**	
Did not finish high school	9 (42.9)
Year 12 or equivalent	5 (23.8)
Non-tertiary education	5 (23.8)
Tertiary education	2 (9.5)
**Housing Structure**	
Family with dependent children	3 (14.3)
Couple only	1 (4.8)
Lone person	5 (23.8)
Group household	12 (57.1)
**Living Situation**	
Homeowner/renter/resident of social housing	10 (47.6)
Resident of assisted living facility/residential care accommodation	4 (19.1)
Resident of short-term emergency care	7 (33.3)
**Number of people living in usual residence**	
1–2 people	6 (28.6)
3–5 people	2 (9.5)
6+people	13 (61.9)
**Meal preparation and frequency**	
Prepare no meals	2 (9.5)
Prepare some meals	11 (52.4)
Prepare most meals	4 (19.1)
Prepare all meals	4 (19.1)

**Table 3:** Participant’s food security scores (6-item USDA FSM) (*n* = 21).

Food Security Scores	Pre *n* (%)	Post *n* (%)
**Individual 6-item scores**		
Ran out of food	13 (61.9)	9 (42.9)
Couldn’t afford healthy meals	12 (57.1)	14 (66.7)
Adults cut size or skipped meals	7 (33.3)	3 (14.3)
Frequency adults cut/skipped meals	5 (23.8)	2 (9.5)
Ate less than thought should	8 (38.1)	5 (23.8)
Hungry but didn’t eat	4 (19.0)	2 (9.5)
**Level of severity scores**		
High or marginal food security (0–1)	8 (38.1)	12 (57.1)
Low or very low food security total (2–6)	13 (61.9)	9 (42.9)
- Low food security (2–4)	10 (47.6)	7 (33.4)
- Very low food security (5–6)	3 (14.3)	2 (9.5)

**Table 4:** Participant’s food literacy (*n* = 21).

Food Literacy Measure	Pre Mean (±SD)	Post Mean (±SD)	*p* Value ^1^
*Cooking confidence (Scale of 1–Not confident to 5–extremely confident)*			
Combined average cooking confidence score	3.10 (0.56)	3.76 (0.63)	<0.001 *
Confidence to eat the recommended servings of fruit and vegetables each day	2.67 (0.73)	3.42 (0.93)	0.009 *
Confidence in ability to buy healthy food on a budget	3.19 (1.08)	3.57 (1.61)	0.194
Confidence to cook from basic ingredients	3.33 (0.97)	3.95 (1.12)	0.014 *
Confidence in following a simple recipe	3.29 (0.20)	4.00 (0.22)	0.002 *
Confidence in tasting foods not eaten before	3.05 (1.16)	3.86 (0.96)	0.005 *
Confidence in preparing and cooking new foods and recipes	3.05 (0.67)	3.76 (0.89)	0.001 *
*Food behaviours on a scale of 0 (never) to 3 (always)*			
Combined average food behaviours score	1.39 (0.62)	1.76 (0.70)	0.006 *
Look for low-salt food varieties	0.81 (1.03)	1.47 (1.03)	0.007 *
Choose wholemeal or wholegrain bread	1.48 (1.03)	1.95 (1.02)	0.030 *
Read nutrition information panels when shopping	1.05 (0.97)	1.76 (0.94)	0.004 *
Read ingredient list when shopping	1.24 (1.14)	1.57 (0.93)	0.186
Look at price per kilo when shopping	2.05 (0.92)	2.14 (0.96)	0.676
Change recipes to make them healthier	1.34 (1.02)	1.52 (0.87)	0.238
Add salt to food when cooking	1.71 (1.23)	1.67 (0.91)	0.740
Use a shopping list	1.48 (0.98)	2.00 (0.92)	0.012 *
*Nutrition knowledge (score 0–5)*			
Average total nutrition knowledge score	2.57 (0.98)	3.09 (0.89)	0.033 *

^1^ Wilcoxon signed-rank test based on comparison of medians uses the median as a measure for central tendency. *p* values displayed are based on medians not the means displayed. * Statistically significant value (*p* value < 0.05).

**Table 5:** Average intake of selected food and beverages (no. serves/day) (*n* = 21).

Foods and Beverages	Pre Mean (±SD)	Post Mean (±SD)	*p* Value ^1^
*Fruit, vegetables, and water*			
Vegetables	1.71 (1.07)	2.36 (1.45)	0.043 *
Fruit	1.55 (0.97)	1.83 (1.08)	0.209
Water	5.05 (3.22)	4.90 (3.10)	0.860
*Discretionary beverages*			
Sugar-sweetened beverages	1.24 (1.46)	0.83 (0.89)	0.017 *
*Discretionary foods*			
Overall discretionary foods	1.72 (1.22)	1.28 (1.05)	0.140
Potato crisps or salty snack foods	0.28 (0.29)	0.16 (0.22)	0.011 *
Chocolate or lollies	0.54 (0.75)	0.47 (0.72)	0.805
Cake, doughnuts, sweet biscuits	0.38 (0.42)	0.27 (0.33)	0.184
Pies, pasties, sausage rolls	0.36 (0.29)	0.24 (0.32)	0.155
Fast foods (e.g., McDonalds, KFC)	0.11 (0.12)	0.09 (0.17)	0.050
Pizza (shop bought or homemade)	0.06 (0.62)	0.06 (0.42)	0.661

^1^ Wilcoxon signed-rank test uses the median as a measure for central tendency. *p* values displayed are based on medians not the means displayed. * Statistically significant (*p* value < 0.05).

**Table 6:** Demographics of NEST interview participants (*n* = 17).

Category	*n* (%)
**Gender**	
Male	11 (64.7)
Female	6 (35.3)
***Age***	
18–34 years	3 (17.7)
35–54 years	9 (52.9)
55–74 years	4 (23.5)
75+ years	1 (5.9)
**Nationality**	
Australian	12 (70.6)
Non-Australian	5 (29.4)
**Primary Language**	
English	17 (100)
Non-English	0 (0)
**Housing Status**	
Housing Commission	6 (35.3)
Rehabilitation Centre	5 (29.4)
Renting	4 (23.5)
Homeowner	2 (11.8)
**Children in home**	
0	16 (94.1)
1–5	1 (5.9)
**Other adults in home**	
0	4 (23.5)
1–5	3 (17.7)
6+	10 (58.8)
**Weekly household income**	
<$575	11 (64.7)
$575–865	2 (11.7)
$865–1150	1 (5.9)
>$1150	1 (5.9)
Prefer not to say	2 (11.8)
**Food security level**	
High or marginal food security (0–1)	9 (53.0)
Low or very low food security total (2–6)	8 (47.0)
Low food security (2–4)	4 (23.5)
Very low food security (5–6)	4 (23.5)

## Supplementary Materials

The following are available online at https://www.mdpi.com/2072-6643/12/8/2197/s1, S1: COREQ Checklist; S2: Pre-Post Survey; S3: Interview Guide and Field Notes; S4: Summary of NEST Program.
