# Supplementary material for: The Role of a Food Literacy Intervention in Promoting Food Security and Food Literacy—OzHarvest’s NEST Program
Source: Nutrients. 2020 Jul 23;12(8):2197. doi: 10.3390/nu12082197 (PMC7468773; doi:10.3390/nu12082197)
Supplement: Supplementary file 1 [file nutrients-12-02197-s001.zip › S2 - Nest Pre-Post Survey.pdf]

PRE-POST PARTICIPANT SURVEY

By completing this survey you will help us to measure the impact of the program, so that we can continue to improve it. We hope you enjoy the NEST program!

UNIQUE ID

To make sure that this survey remains anonymous, we won't be asking for any names or contact details. But we have 4 questions for you to answer, so we can give your survey its own unique ID.

1. **What is the first letter of the city/town of this NEST program? (e.g. Melbourne = M)** * *Mark only one.*

☐ (M) Melbourne ☐ (N) Newcastle ☐ (B) Brisbane

☐ (S) Sydney ☐ (G) Gold Coast ☐ (A) Adelaide ☐ (C) Canberra

2. **What number represents the month you were born? (e.g. January = 01)** * *Mark only one.*

☐ 01 ☐ 04 ☐ 07 ☐ 10

☐ 02 ☐ 05 ☐ 08 ☐ 11

☐ 03 ☐ 06 ☐ 09 ☐ 12

3. **What is the first letter of your mother's first name? (e.g. Susan = S, if unknown use X)** * *Mark only one.*

A ☐ E ☐ I ☐ M ☐ Q ☐ U ☐ Y ☐

B ☐ F ☐ J ☐ N ☐ R ☐ V ☐ Z ☐

C ☐ G ☐ K ☐ O ☐ S ☐ W ☐

D ☐ H ☐ L ☐ P ☐ T ☐ X ☐

4. What is the first letter of your middle name? (e.g. Ann = A, if you don't have a middle name use X) * *Mark only one.*

A ☐	E ☐	I ☐	M ☐	Q ☐	U ☐	Y ☐
B ☐	F ☐	J ☐	N ☐	R ☐	V ☐	Z ☐
C ☐	G ☐	K ☐	O ☐	S ☐	W ☐	
D ☐	H ☐	L ☐	P ☐	T ☐	X ☐	

PRE OR POST SURVEY

5. Is this your first or last NEST session? * *Mark only one oval.*

- ◯ First session - Pre-program Survey *Skip to question 6.*
- ◯ Last session - Post-program Survey *Skip to question 16.*

DEMOGRAPHIC INFORMATION

6. What is your age? * *Mark only one.*

18-34 years	35-54 years	55-74 years	75+ years

7. What is your gender? * *Mark only one.*

Male	Female	Prefer not to say	Other ____________________

8. Are you of Aboriginal or Torres Strait Islander Origin? * *Mark only one.*

No	Yes, Aboriginal	Yes, Torres Strait Islander

9. What is the postcode of your usual residence/household? *

10. Including yourself how many people live in your usual residence/household? * *Mark only one.*

1	2	3	4	5	6+

11. Which best describes your current employment status? * *Mark only one oval.*

- Full-time employee
- Part-time employee
- Self-employed
- Employed - unpaid worker (e.g. volunteer or family business)
- Unemployed - seeking full time or part-time work
- Not employed - not seeking employment (e.g. pension, disability support pension)

12. Which best describes your current food preparation situation? * *Mark only one oval.*

- I prepare/cook ALL food/meals for myself and/or others
- I prepare/cook MOST food/meals for myself and/or others
- I prepare/cook SOME food/meals for myself and/or others
- I prepare/cook NO food/meals for myself and/or others

13. What is the highest level of education that you have completed? * *Mark only one.*

- Never attended school
- Year 8 or below
- Year 9 or equivalent
- Year 10 or equivalent
- Year 11 or equivalent
- Year 12 or equivalent
- Tafe Certificate 1,2,3,4
- Advanced Diploma or Diploma Level
- Bachelor's degree Level
- Graduate Diploma and Graduate Certificate Level
- Postgraduate Degree Level
- Other

14. Which best describes your current household structure?

* *Mark only one oval.*

- Couple family with dependent children
- One parent family with dependent children
- Couple only
- Multiple family household
- Lone person
- Group household (e.g. community housing, rehabilitation housing etc)
- Other

15. Which best describes your current living situation?

* *Mark only one oval.*

- ◯ Living in a house/apartment/flat that I (own/rent/social public housing)
- ◯ Living in an assisted living facility/residential care/temporary accommodation/caravan
- ◯ Living in a short-term emergency accommodation/crisis shelter/transitional housing
- ◯ Living in varied places including couch surfing/tents/sleeping rough
- ◯ Other: ______________________

Skip to question 27.

OVERALL SATISFACTION WITH NEST PROGRAM

16. Tick the session(s) which you have attended * *Tick all that apply.*

- ☐ Module 1: Eat for Variety
- ☐ Module 2: Eat for Wellbeing
- ☐ Module 3: Eat for Balance
- ☐ Module 4: Eat for the Environment
- ☐ Module 5: Eat for Choice
- ☐ Module 6: Eat for Life

17. On a scale of 1-5, how would you rate your overall experience of the NEST program? *

Mark only one.

Not good	1	2	3	4	5	Very good

18. What did you like most about the NEST program? **Mark only one oval..*

- ◯ Education
- ◯ Cooking
- ◯ Games and activities
- ◯ Cookbooks, handouts, resources
- ◯ I liked everything about the program
- ◯ I did not like anything about the program
- ◯ Other:

19. What did you like least about the NEST program? * *Mark only one oval.*

- ⬭ Education
- ⬭ Cooking
- ⬭ Games and activities
- ⬭ Cookbooks, handouts, resources
- ⬭ I liked everything about the program
- ⬭ I did not like anything about the program

20. On a scale of 1-5, how likely would you recommend the program to a friend or family member? *
Mark only one.

Not likely	1	2	3	4	5	Very likely

21. Repeating this survey in 6 months time can provide valuable insights into sustained changes in food knowledge, attitudes, beliefs, skills, and behaviours. This can help us to improve future programs. Do you consent to being contacted in the future for a 6-month follow up? * *Mark only one oval.*

- ⬭ Yes *Skip to question 22.*
- ⬭ No *Skip to question 27.*

CONTACT DETAILS:

You have ticked 'yes' to be followed up in 6 months, please print your full name to indicate your consent, the date of consent, and please provide us with your contact details.

22. Full name * ________________________________

23. Agency name * ________________________________

24. Date of consent * ________________________________

25. Email address * ________________________________

26. Contact Phone Number * ________________________________

Skip to question 27.

FOOD LITERACY

27. How many servings of fruit should you eat every day for good health? **Mark only one.*

1	2	3	4	5	6	Serves per day

28. How many servings of vegetables should you eat every day for good health? **Mark only one.*

1	2	3	4	5	6	Serves per day

NUTRITION PANEL

Please use the Nutrition Information Panel to answer the following questions:

Nutrition Information

Servings per package – 16
Serving size – 30g (2/3 cup)

	Per serve	Per 100g
**Energy**	**432kJ**	**1441kJ**
**Protein**	2.8g	9.3g
**Fat**		
Total	0.4g	1.2g
Saturated	0.1g	0.3g
**Carbohydrate**		
Total	18.9g	62.9g
Sugars	3.5g	11.8g
**Fibre**	6.4g	21.2g
**Sodium**	65mg	215mg

Ingredients: Cereals (76%) (wheat, oatbran, barley), psyllium husk (11%), sugar, rice, malt extract, honey, salt, vitamins.

Figure 1: Eat for Health - Eating well food label

29. What is the main ingredient in the product? * *Mark only one.*

- ☐ Sugar
- ☐ Honey
- ☐ Rice
- ☐ Salt
- ☐ Cereals
- ☐ Not sure

30. In this product is there more sugar or rice? *

Mark only one.

More sugar	More rice	Don't know

31. In 100 grams of this product, how many grams of sugar are there? * *Mark only one.*

- ☐ 11.8 grams
- ☐ 1.2 grams
- ☐ 21.2 grams
- ☐ 1.3 grams

CONFIDENCE AND SELF-EFFICACY

32. On a scale of 1-5, how confident are you that you can eat the recommended number of servings of fruit each day? * *Mark only one number.*

Not at all confident	1	2	3	4	5	Extremely confident

33. On a scale of 1-5, how confident are you that you can eat the recommended number of servings of vegetables each day? * *Mark only one number.*

Not at all confident	1	2	3	4	5	Extremely confident

34. On a scale of 1-5, how confident are you that you can buy healthy foods on a budget? * *Mark only one number.*

Not at all confident	1	2	3	4	5	Extremely confident

35. On a scale of 1-5, how confident do you feel to follow a simple recipe? * *Mark only one number.*

Not at all confident	1	2	3	4	5	Extremely confident

36. On a scale of 1-5, how confident do you feel about following a simple recipe? * *Mark only one number.*

Not at all confident	1	2	3	4	5	Extremely confident

37. On a scale of 1-5, how confident do you feel about tasting foods that you have not eaten before? * *Mark only one number*

Not at all confident	1	2	3	4	5	Extremely confident

38. On a scale of 1-5, how confident do you feel about preparing and cooking new foods and recipes? * *Mark only one number.*

Not at all confident	1	2	3	4	5	Extremely confident

FOOD BEHAVIOURS

39. How often do you look for low-salt varieties of foods? * *Mark only one.*

Always	Often	Sometimes	Never

40. How often do you choose wholemeal/wholegrain bread? * *Mark only one.*

Always	Often	Sometimes	Never

41. How often do you read the Nutrition Information Panel on food items when you go shopping? * *Mark only one.*

Always	Often	Sometimes	Never

42. How often do you read the ingredients list on food items when you go shopping? **Mark only one.*

Always	Often	Sometimes	Never

43. How often do you look at the price per kilo when shopping? * *Mark only one.*

Always	Often	Sometimes	Never

44. How often do you change recipes to make them healthier? **Mark only one.*

Always	Often	Sometimes	Never

45. How often do you add salt to food during cooking? * *Mark only one.*

Always	Often	Sometimes	Never

46. How often do you use a shopping list? * *Mark only one.*

Always	Often	Sometimes	Never

VEGETABLES & FRUIT

47. About how many serves of VEGETABLES do you usually eat per day? *Do not include hot chips or fried potato*. **Mark only one oval.*

- I don't eat vegetables
- Less than 1 serve per day
- 1 serve per day
- 2 serves per day
- 3 serves per day
- 4 serves per day
- 5 serves per day
- 6 or more serves per day

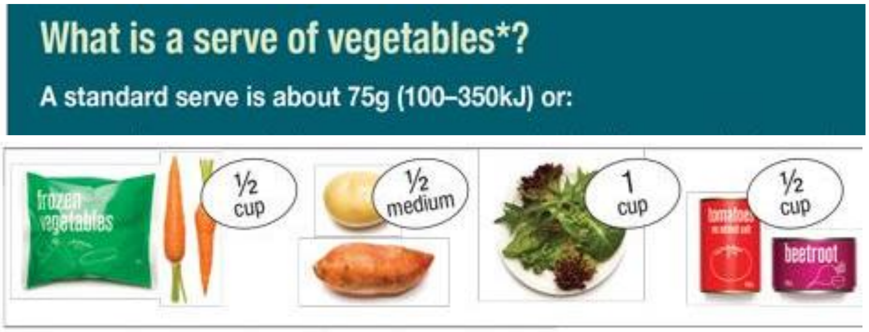

*With canned varieties, choose those with no added salt

48. About how many serves of FRUIT do you usually eat per day? Do not include fruit juice or fruit drink. * *Mark only one oval.*

- 1 don't eat fruit
- Less than 1 serve per day
- 1 serve per day
- 2 serves per day
- 3 serves per day
- 4 serves per day
- 5 serves per day
- 6 or more serves per day

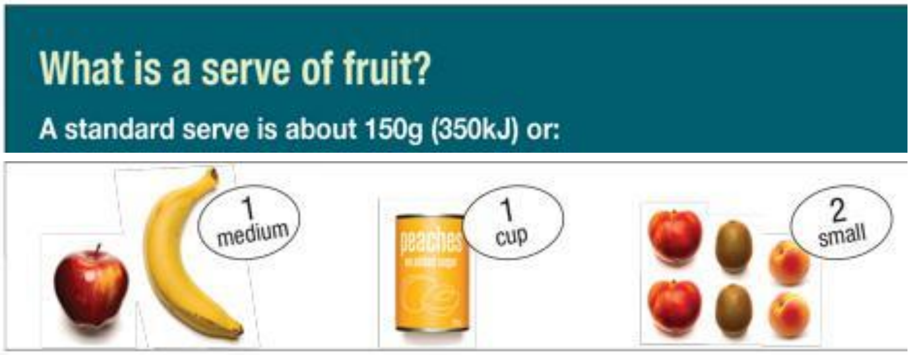

KEY DISCRETIONARY FOODS

49. In the past 6 weeks how often have you eaten potato crisps or salty snack foods? * *Mark only one.*

Never or less than once/ month	1-3 times/ month	Once/ week	2-4 times/ week	5-6 times/ week	Once/ day	2-3 times/ day	4-5 times/ day	6 or more times/ day

50. In the past 6 weeks how often have you eaten chocolate or lollies? * *Mark only one.*

Never or less than once/ month	1-3 times/ month	Once/ week	2-4 times/ week	5-6 times/ week	Once/ day	2-3 times/ day	4-5 times/ day	6 or more times/ day

51. In the past 6 weeks how often have you eaten cake, doughnuts, sweet biscuits? * *Mark only one.*

Never or less than once/ month	1-3 times/ month	Once/ week	2-4 times/ week	5-6 times/ week	Once/ day	2-3 times/ day	4-5 times/ day	6 or more times/ day

52. In the past 6 weeks how often have you eaten pies, pasties, or sausage rolls? * *Mark only one.*

Never or less than once/ month	1-3 times/ month	Once/ week	2-4 times/ week	5-6 times/ week	Once/ day	2-3 times/ day	4-5 times/ day	6 or more times/ day

53. In the past 6 weeks how often have you eaten fast foods (e.g. McDonalds, KFC)? * *Mark only one.*

Never or less than once/ month	1-3 times/ month	Once/ week	2-4 times/ week	5-6 times/ week	Once/ day	2-3 times/ day	4-5 times/ day	6 or more times/ day

54. In the past 6 weeks how often have you eaten pizza (all kinds including takeaway and homemade)? **Mark only one.*

Never or less than once/ month	1-3 times/ month	Once/ week	2-4 times/ week	5-6 times/ week	Once/ day	2-3 times/ day	4-5 times/ day	6 or more times/ day

SUGAR-SWEETENED BEVERAGE CONSUMPTION

55. How many cups of soft drink, cordials, sports drinks or caffeinated energy drinks do you usually drink each day? * *Mark only one oval.*

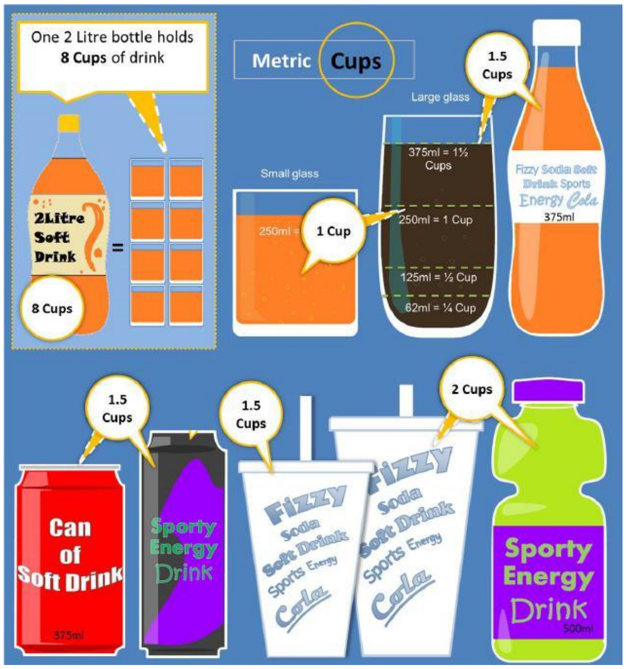

- I don't drink sugar-sweetened drinks
- I choose diet or no sugar drinks
- Less than 1 cup per day
- 1 cup per day
- 2 cups per day
- 3 cups per day
- 4-5 cups per day
- 6-7 cups per day
- 8-9 cups per day
- 10 or more cups per day

WATER CONSUMPTION

1/2 a cup of water = 1 serve

A 600ml bottle of water = 5 serves

A 1L bottle of water = 8 serves

56. About how many serves of PLAIN WATER in total do you usually drink each day? **Mark only one oval.*

- I don't drink plain water
- Less than 1 serve per day
- 1 serve per day
- 2 serves per day
- 3 serves
- 4-5 serves
- 6-7 serves
- 8-9 serves
- 10 or more serves

FOOD SECURITY

These next questions are about the food eaten in your household in the last 6 weeks and whether you were able to afford the food you need.

57. "The food that (I/we) bought just didn't last, and (I/we) didn't have money to get more." In the last 6 weeks was this: **Mark only one.*

Often true	Sometimes true	Never true	Don't know

58. "(I/we) couldn't afford to eat balanced meals." In the last 6 weeks was this: * *Mark only one.*

Often true	Sometimes true	Never true	Don't know

59. In the last 6 weeks, did you/or other adults in your household ever cut the size of your meals or skip meals because there wasn't enough money for food? * *Mark only one.*

Yes *Skip to question 68.*	No *Skip to question 69.*	Don't know *Skip to question 69.*

60. How often did this happen? **Mark only one.*

Almost every month	Some months but not every month	Only 1 or 2 months	Don't know	Not applicable

Skip to question 69

61. In the last 6 weeks, did you ever eat less than you felt you should because there wasn't enough money for food? *Mark only one.*

Yes	No	Don't know

62. In the last 6 weeks, were you ever hungry but didn't eat because there wasn't enough money for food? *Mark only one.*

Yes	No	Don't know

THANK YOU FOR COMPLETING OUR SURVEY!
