# Supplementary material for: The Role of a Food Literacy Intervention in Promoting Food Security and Food Literacy—OzHarvest’s NEST Program
Source: Nutrients. 2020 Jul 23;12(8):2197. doi: 10.3390/nu12082197 (PMC7468773; doi:10.3390/nu12082197)
Supplement: Supplementary file 1 [file nutrients-12-02197-s001.zip › S3 - Interview Guide and Field Notes.pdf]

Interview Guide and Field Notes

Study Title: The role of nutrition education and food literacy interventions to address household food insecurity.

Researcher: Elisha West

Date: _________________

Time: ______ **to** _______

Participant Name: __________________

Participant ID: ___ ___ ___ ____ (see 4 questions at end)

NB: Remind participants to not to provide any identifying information about themselves or a third party during the interview

Below provides the outline of the interview guide and content to be explored:

1. Can you tell me a little bit about yourself? *Prompt: family, hobbies/interests, where they grew up etc…*

2. Can you tell me a bit about the food you eat? *Prompt: Where you source your food from? How you shop? How you cook?*

3. So, you were involved in the NEST program recently, how long ago was that?

4. Why did you decide to sign up for the NEST program?

5. Overall, how did you find the program?

6. In the NEST program lots of different changes were suggested on ways to shop and cook healthy food on a budget, what are some suggested changes that you remember from the program?

7. Have you made any of these changes to the way you shop or cook food since completing the NEST program? *Prompt - If yes how? If not is there a reason for this?*

8. In the NEST program there were also changes suggested on ways to eat, what are some suggested changes that you remember from the program?

9. Have you made any of these changes to the way you eating since completing the program? *Prompt - If yes how? If not is there a reason for this?*

10. In the NEST program, you set goals each week, do you recall any of the goals that you set? *Prompt – If yes, what were they?*

11. Have you stuck with any of these goals since completing the program? *Prompt - If yes how? If not is there a reason for this?*

12. Have any members of your family or household changed their eating since the NEST program? Please explain.

13. Has anything else changed for you as a result of the NEST program?

14. Has anything else changed for your family or household as a result of the NEST program?

15. You mentioned <insert changes/no changes>, what barriers did you face when trying to stick with any changes suggested in the NEST program? Please explain.

16. You mentioned <insert changes/no changes>, did anything help you/make it easier for you to stick with any changes suggested in the program? Please explain.

17. Have you continued to use any of the NEST program materials? *Prompt - Which ones? How often?*

18. Have you shared the program materials with anyone else? *Prompt – If yes which ones and who?*

19. What is the most important thing you remember from the program?

20. Thank you for your time. Is there anything else you'd like to add about what we've discussed today?

Demographic and food security questions post-interview

14. How old are you? ____________

15. What is your nationality (as shown on your passport)? ________________

16. What is your gender?

- ☐ Male
- ☐ Female
- ☐ Other

16. What language do you MAINLY speak at home? __________________

17. What is your current housing status (for e.g. renting, social housing, assisted living)?

____

18. Other than you, how many adults, and how many children usually live in your household?

Number of Adults: ____________ Number of Children:___________

19. What is your household income per week, including all additional payments such as Centrelink?

- ☐ Less than $575
- ☐ $575-865
- ☐ $865-1150
- ☐ More than $1150
- ☐ Prefer not to say

Finally, these next questions include statements about the food eaten in your household in the last 12 months.

1. "The food that (I/we) bought just didn't last, and (I/we) didn't have money to get more."
Was that often, sometimes, or never true for (you/your household) in the last 6 weeks?

- Often
- Sometimes
- Never true

2. "(I/we) couldn't afford to eat balanced meals."
Was that often, sometimes, or never true for (you/your household) in the last 6 weeks?

- Often
- Sometimes
- Never true

3a. "In the last 6 weeks, did (you/you or other adults in your household) ever cut the size of your meals or skip meals because there wasn't enough money for food?"

- Yes
- No (Skip 22a)
- Don't Know (Skip 22a)

3b. [IF YES ABOVE] How often did this happen—almost every month, some months but not every month, or in only 1 or 2 months?

- Almost every month
- Some months but not every month
- Only 1 or 2 months

4. In the last 6 weeks, did you ever eat less than you felt you should because there wasn't enough money for food?

- Yes
- No
- Don't Know

5. In the last 6 weeks, were you ever hungry but didn't eat because there wasn't enough money for food?

- Yes
- No (Skip 22a)
- Don't Know (Skip 22a)

1. What is the first letter of the city/town of this NEST program? (e.g. Melbourne = M)
2. What is the first letter of your mother's first name? (e.g. Mary = M, if unknown use X)
3. What number represents the month you were born? (e.g. January = 01)
4. What is the first letter of your middle name? (e.g. Ann = A, if you don't have a middle name use X)

Field Notes

Setting (geographical location and type of setting):

Participants (overall appearance, demeanour, character, baseline non-verbal behaviours):

Interview (participant response to the interview, any changes to questions and why):

Critical Reflection (whole of interview, my performance, bias, and feelings as participant and interviewer, relationship to interviewee):
