# Supplementary material for: The Role of a Food Literacy Intervention in Promoting Food Security and Food Literacy—OzHarvest’s NEST Program
Source: Nutrients. 2020 Jul 23;12(8):2197. doi: 10.3390/nu12082197 (PMC7468773; doi:10.3390/nu12082197)
Supplement: Supplementary file 1 [file nutrients-12-02197-s001.zip › S4 - NEST Program summary.pdf]

OZHARVEST

NEST™

NUTRITION EDUCATION & SKILLS TRAINING

PROGRAM SUMMARY

2020

CREATED BY

Elisha West

Cassie Winter

Irene Sangadi

WITH ASSISTANCE FROM

The team of NEST Coordinators, students, and volunteers

OZHARVEST

Office Address: 854 Lorimer Street, Port Melbourne VIC 3207

T: 03 9999 5070

E: elisha.west@ozharvest.org

W: www.ozharvest.org

NEST PROGRAM SUMMARY

BACKGROUND

OzHarvest is a not-for-profit organisation that rescues surplus food that would otherwise go to waste from restaurants, retailers, food outlets and corporate kitchens, and delivers that good food at no cost to charitable agencies who feed men, women and children that are experiencing poverty and food insecurity.

After 6 years of growing OzHarvest's food rescue operations, our CEO and founder Ronni Kahn, received feedback from our charitable agencies that they were pleased with the beautiful rescued food they were receiving, but many were not sure how to cook or prepare many of the foods they were receiving. As a result, the initial idea of the NEST program was forming in Ronni's mind. She connected with leading Nutritionist Dr. Gina Levy and Paediatric Dietitian Eve Reed to create OzHarvest's NEST Program.

Since its inception in 2012, the NEST program has proudly delivered over 2,050 workshops positively impacting nearly 6,300 Australians around the country. However, the public health industry has evolved significantly over the last several years and now more data is available as it relates to best practices and sustainable programming, evaluation, and management frameworks.

Our original NEST program was wonderful, and since its first pilot in 2012, the NEST team have continued to make evidence-based and innovative improvements to make it a robust program for our participants. As a result, nutritionists Cassie Winter and Elisha West commenced a redevelopment of the NEST program in 2018 with support from Deakin University, the University of Wollongong, and University of New South Wales. The program was piloted in 2019 and will be officially relaunched in 2020.

PROGRAM SUMMARY

OzHarvest's Nutrition Education Skills Training (NEST) program is a 6-week guided public health nutrition program, which aims to improve the nutrition, food literacy, and cooking skills of Australian adults. OzHarvest's NEST Coordinators travel to charitable agencies and organisations each week with all food, equipment, and educational materials required for program delivery. Each 2.5-hour session is delivered to a group of 6-15 adults and integrates a series of hands-on nutrition and food literacy activities, practical cooking lessons, and culminates in the sharing of a meal together.

NEST is aligned with the Australian Dietary Guidelines, and the most recent state/territory-based healthy eating strategies, with significant support from researchers from Deakin University, University of Wollongong, and University of New South Wales. The program is underpinned by behavioural change theory (social cognitive theory) and incorporates best practice and sustainable public health program and evaluation approaches.

CORE & ELECTIVE MODULE OUTLINE

The 6-week program includes 11 modules (5 core modules and 6 elective modules). NEST coordinators, agencies, and participants collaboratively select which of the six elective modules are most appropriate for the NEST participant group. This module is delivered in the 5th week of the NEST program.

CORE MODULES

MODULE 1: EAT FOR VARIETY

Outline:
- What is healthy eating?
- Choosing a variety of foods from the 5 food groups
- Increasing fruit and vegetables

MODULE 2: EAT FOR WELLBEING

Outline:
- Key nutrients and healthier options within the five food groups
- Portion vs. serve size

MODULE 3: EAT FOR BALANCE

Outline:
- Discretionary foods as part of a balanced diet
- Label reading
- Identifying foods high in sugar, salt and fats

MODULE 4: EAT FOR THE ENVIRONMENT

Outline:
- Tips to fight food waste
- Safe food handling and storage methods

MODULE 5: EAT FOR THE CHOICE

Agencies choose ONE of the following:
- Eating for Diabetes Management
- Eat to Move (Physical Activity)
- Eating for Infants and Young Children
- Eat from the Supermarket
- Eat Healthy when Eating Out
- Eat for Pregnancy and Lactation

MODULE 6: EAT FOR LIFE

Outline:
- Bringing it all together
- Comparing the price of healthy vs. less healthy foods
- Healthy eating on a budget

OPTIONS FOR MODULE 5: EAT FOR CHOICE

EAT FOR DIABETES MANAGEMENT

Outline:
- Food as carbohydrates, protein, and fats
- 'Free foods'
- Type, amount, and frequency of carbohydrates

EAT TO MOVE

Outline:
- Importance of physical activity
- Australian guidelines
- Active swaps

EAT FROM THE SUPERMARKET

Outline:
- Health claims, key nutrients and healthier options in different sections of the supermarket

HEALTHY CHOICES WHEN EATING OUT

Outline:
- Dining out tips and traps
- Energy dense nutrient poor foods
- Healthier swaps

EATING FOR INFANTS AND YOUNG CHILDREN

Outline:
- Introducing solids and first foods
- Texture progression
- Parent-child division of responsibility during mealtimes

EAT FOR PREGNANCY AND BREASTFEEDING

Outline:
- Healthy eating guidelines for pregnancy and lactation
- Supplement use

GOAL & AIMS

The goal of NEST is to create positive, long-term change in the dietary behaviours and food literacy of low socio-economic individuals.

The aims of the program are:

- To improve participants' food literacy in terms of knowledge, attitudes, beliefs, skills and behaviours related to healthy eating and its accessibility.
- To increase consumption of core foods aligned with dietary guidelines.
- To decrease consumption of key discretionary (energy-dense, nutrient poor) foods and drinks.
- To reduce household food insecurity
- To increase social engagement and connections in the community
- To upskill agency staff on healthy eating, food insecurity, and how to support behavioural change in their clients

SMART OBJECTIVES

The Program's objectives, key program strategies, and impact evaluation:

Objective 1: Reduce consumption of discretionary, energy dense, and nutrient poor foods and drinks, in at least 20% of participants.

Objective 2: Increase consumption of core foods aligned with dietary guidelines (especially fruit and vegetable consumption) in at least 30% of participants.

Objective 3: Increase food and nutrition knowledge for healthy eating on a budget in at least 80% of participants.

Objective 4: Increase skills and self-efficacy (confidence) for healthy eating on a budget in at least 80% of participants.

Objective 5: Reduce perceived barriers to healthy eating such as attitudes, beliefs, and behaviours in at least 50% of participants.

Objective 6: Reduce household food insecurity in at least 10% of participants.

Objective 7: Increase social engagement and connections in the community.

Objective 8: Upskill charitable agency staff on healthy eating, food insecurity and how to support behaviour change in their clients, in at least 50% of agencies.

Objective 9: 90% of NEST participants are likely or very likely to recommend the NEST program to others.

Objective 10: 90% of NEST participants had a good or very good experience at the NEST program.

NEST RESOURCES

EVERYDAY COOKBOOK

Over the six-week program, participants will work through creating recipes from the OzHarvest Everyday Cookbook.

The cookbook is packed full of 30 easy, low-cost, healthy and delicious step by step photo-based recipes (most of which cost less than $2.00 per serve!).

All participants are provided with their own copy to keep at the end of the program.

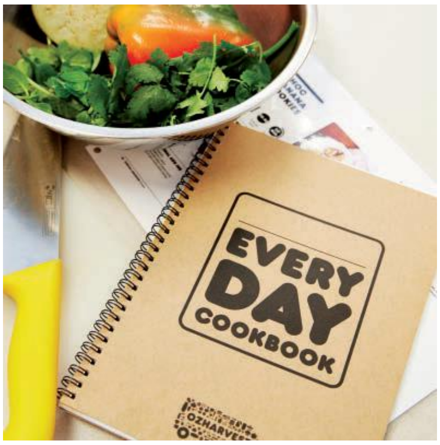

GOAL SETTING DIARY

At each NEST session, our team of nutritionists and dietitians work with participants to create personal SMART goals for the week in their Goal Diary relating to the week's topic.

These goals are revisited and ticked off weekly and helps introduce accountability and maintain motivation for longer term behaviour change.

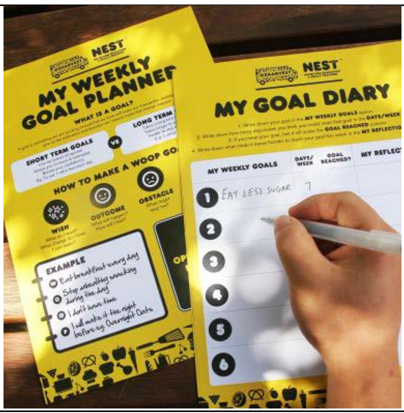

PARTICIPANT TOOLKIT

At the end of the program, in addition to the cookbook, each participant will take home a practical toolkit filled with resources to help remind them of the program's key learnings:

- Portion Plate Guide
- Understanding food labels factsheet
- Tips for shopping on a budget and healthier supermarket shopping and eating out
- Healthy Food and Drinks Pocket Card

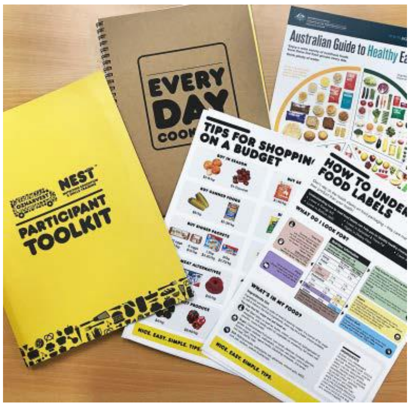

AGENCY TOOLKIT

NEST wants to support the agencies in encouraging healthier eating behaviours taught in the program long after the program ends.
That's why we've created the agency toolkit which will be provided at the end of the program to include the following:

- Swap and Switch Poster
- Food Safety Poster
- Australian Guide to Healthy Eating Poster
- Healthy Plate Poster
- OzHarvest Everyday Cookbook

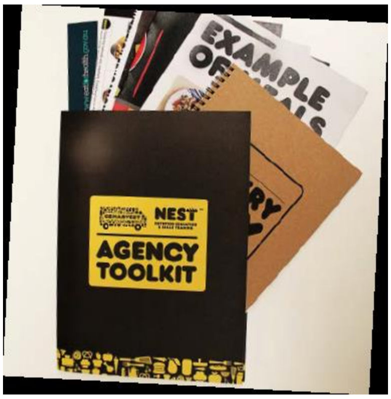

PROGRAM EVALUATION FRAMEWORK

OzHarvest has made a significant investment to update and upgrade the NEST program. Thus, the program design is now evidence based, the teaching tools and methods appropriate for a low socio-economic adult population and participant demographic and impact data are collected using a validated survey.

On the back of a successful pilot phase, NEST continues to embed best practices in implementation from a ***process and outcome evaluation*** perspective so that the program can be monitored, sustained and/or adapted in a systematic way to ensure program sustainability and integrity.

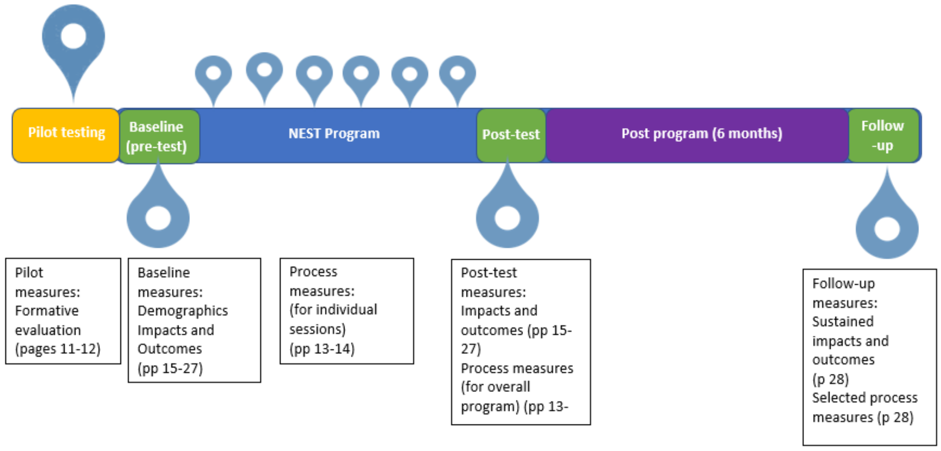

OUTCOME EVALUATION

The program's evaluation framework and logic model were developed in collaboration with researchers from Deakin University's Institute of Physical Activity and Nutrition (IPAN). The program utilises validated research methods to measure the participant's dietary behaviours and food literacy at baseline (pre-program), at the completion of the program, and a 6-month follow up is also conducted to measure the effectiveness of the program in relation to the program's objectives.

LOGIC MODEL FOR HYPOTHESISED PATHWAYS (MEDIATORS) OF EFFECTS OF NEST PROGRAM FOR PARTICIPANTS:

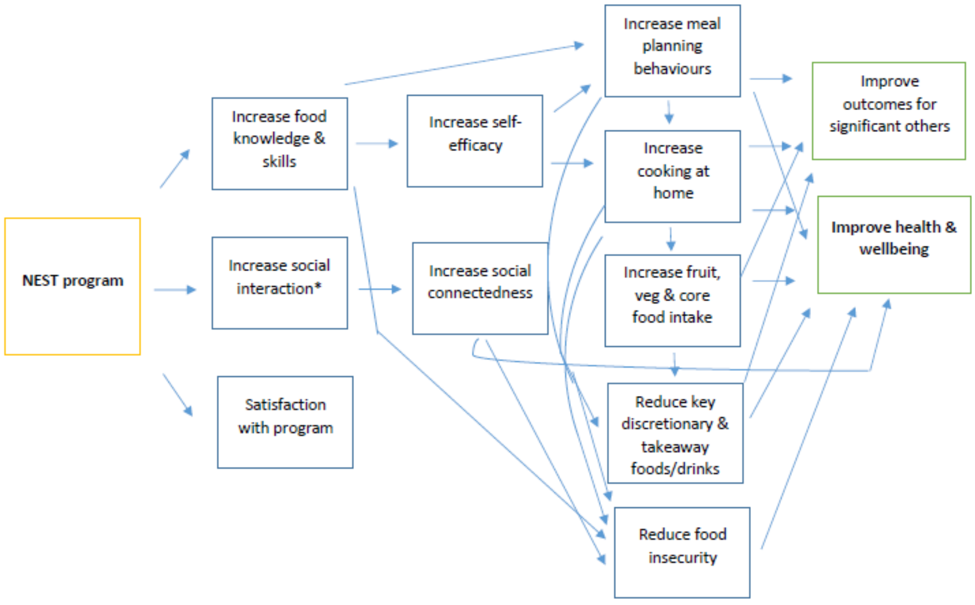

PROCESS EVALUATION

The NEST program's process evaluation tools and activities are based on the need to balance implementation fidelity with adaptability. This ensures NEST is being delivered as intended to produce quality, consistent outcomes; or facilitate the adaptation of program delivery to ensure the latter. Ongoing structured evaluation has been embedded into the program for effective program management, for ensuring program efficiency and relevance, and most importantly it ensures program accountability. The below image represents the ethos behind this evaluation framework, that balances the need for fidelity and adaptability.

From: The dynamic sustainability framework: addressing the paradox of sustainment amid ongoing change

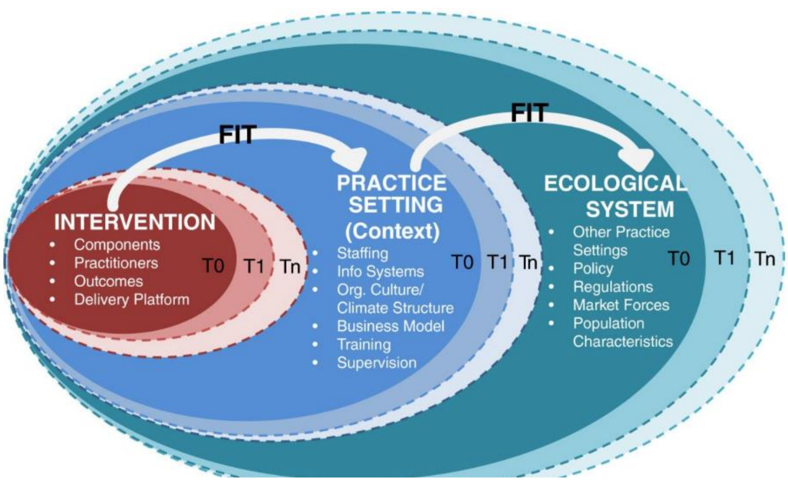

This evaluation of the process allows OzHarvest to determine when efficiencies, updates, and additional training are required. OzHarvest also conducts process evaluation through NEST Facilitators by recording the number of workshops, workshop costs, numbers of participants, and numbers of charitable agencies involved.

ADDITIONAL INFORMATION

How does the NEST program work?

Each of the sessions are held at the same day and time each week, and run for 2.5 hours. The workshop is delivered through fun interactive activities, followed by cooking and sharing a meal together, with any leftovers packaged up for people to take home.

How much does it cost?

The cost for a six week, 15-hour program for 6-15 participants is $3,150+ GST which includes:

- All food and equipment
- Travel costs
- Nutrition trained NEST Coordinator
- OzHarvest volunteer support
- One agency toolkit and a toolkit for every participant

If you have a limited budget, please don't be put off by the full cost – let us know what your budget is, and we can discuss what we can do to help you.

Is the program just for adults?

At the moment, all materials for the program are designed for adults (18+ years).

Do we need the same participants every week?

Yes, as we will build on from discussions and goals each week, we need the same participants each week for the full six weeks.

What do participants need to bring/wear?

Participants do not need to bring anything. We provide all the equipment, ingredients, and aprons. Please ensure participants wear comfortable, practical clothing, and closed-toed shoes for safety in the kitchen.

We don't have a proper kitchen, are you still able run NEST at our agency?

Don't worry, the minimum requirement is a sink with running water, a fridge, and some bench space (or trestle tables!) and we will take care of the rest. We bring portable frypans and all cooking utensils with us and there are plenty of recipes in the cookbook that don't require an oven! If you are a new agency, our NEST Coordinator will also conduct a site visit to assess that the space is suitable before confirming a booking.

Is the NEST program accessible for people with a disability or special needs?

We can accommodate participants with a disability or special needs please contact us to discuss their requirements prior to booking. Participants who require a carer due to special needs must ensure the same carer attends all sessions for health and safety purposes. It is preferable for a person with special needs to arrive 15 minutes before the first class to ensure that the NEST team have adequate time to discuss any issues that may arise for the participant during the course.

For more information: www.ozharvest.org/what-we-do/nest-nutrition-education/
