# Supplementary material for: The Role of a Food Literacy Intervention in Promoting Food Security and Food Literacy—OzHarvest’s NEST Program
Source: Nutrients. 2020 Jul 23;12(8):2197. doi: 10.3390/nu12082197 (PMC7468773; doi:10.3390/nu12082197)
Supplement: Supplementary file 1 [file nutrients-12-02197-s001.zip › S1 - COREQ 32 Item Check List .pdf]

COREQ 32 Item Check List

Allison Tong, Peter Sainsbury, Jonathan Craig, Consolidated criteria for reporting qualitative research (COREQ): a 32-item checklist for interviews and focus groups, International Journal for Quality in Health Care, Volume 19, Issue 6, December 2007, Pages 349–357, https://doi.org/10.1093/intqhc/mzm042

Domain 1: Research Team and Reflexivity

Personal Characteristics

1. Interviewer/facilitator - Which author/s conducted the interview or focus group?
Elisha West

2. Credentials - What were the researcher's credentials? E.g. PhD, MD

- BA(Hons) (Health Education and Sociology)
- Grad Cert. Hum Nutr.
- Masters Human Nutrition (current student)

3. Occupation - What was their occupation at the time of the study?
Elisha West's occupation at the start of the study was the Nutrition Education Programs Officer of OzHarvest. Towards the end of the study write up, she was promoted to National NEST Program Manager. In both roles she was involved in the funding, management, and development of the NEST nutrition education and food literacy intervention.

4. Gender - Was the researcher male or female?
Female

5. Experience and training - What experience or training did the researcher have?

- Previous experience conducting focus groups and interviews with people experiencing food insecurity.
- NVIVO Qualitative Software Analysis Training
- At time of research project, was in late stages of completing M.Nutr. where principles of qualitative research were covered in detail.

Relationship with participants

6. Relationship established - Was a relationship established prior to study commencement?

- During the intervention period, I visited 3 (of 7) services (Champion – Temple Society, Dreambuilders Transformations, and Servants Community Housing) to observe the program being delivered in my officer role in week 1 of the 6 week programs.
- The staff and participants at services where NEST was delivered had a pre-existing professional relationship with me as I booked some of the programs with them.

7. Participant knowledge of the interviewer - What did the participants know about the researcher? e.g. personal goals, reasons for doing the research

- Some of the study participants may have been aware of me prior to the interview and as a result, consent to participate in the study might risk being influenced by the researcher's perceived position of power. To mitigate any inadvertent coercion or pressure that may arise from the student researcher's position and professional roles, a carefully thought out recruitment process was devised.

- Participants were aware that I am dedicated to improving the NEST program and to see participants of the program benefit from it, they understood that these were the reasons behind me doing this research.
- Ethics approval had been granted, participants reviewed the participant information documentation prior to giving their written informed consent to be involved.

8. Interviewer characteristics - What characteristics were reported about the interviewer/ facilitator? e.g. Bias, assumptions, reasons and interests in the research topic

I am employed by the not for profit organisation OzHarvest, and therefore I was involved in the development and delivery of the NEST program. OzHarvest collaborated with the Institute for Physical Activity and Nutrition (IPAN) at Deakin University, whom provided recommendation reports on the NEST program's content and evaluation framework. The principal investigator and associate investigator were involved in developing the recommendation reports. This means there is potential or perceived conflict of interest.

This conflict was managed by:

- Investigators declaring on the plain language statement their affiliations* informing potential participants of the investigator's affiliations
- Investigators declaring on the scientific publication(s) their affiliations informing audiences of the investigator's affiliations
- Investigators will report the study's methods, data and analysis in a transparent manner as is expected of scientific research and peer review will help to ensure this transparency and fair reporting and analysis

There are no financial COIs to declare, nor any other conflicts.

I have a strong understanding of the lived experience of food insecurity as a result of working with people experiencing food insecurity through my work at OzHarvest, it was essential that I didn't let this previous experience cause me to create assumptions on what participants were communicating during the interview. I made sure that I asked for clarification and for further explanation even if I perceived what the participants were explaining to ensure that the interview transcripts provided adequate responses to the questions, and that there were no gaps in the transcripts as a result of my assumptions.

Domain 2: Study Design

<u>Theoretical framework</u>

9. Methodological orientation and Theory - What methodological orientation was stated to underpin the study? e.g. grounded theory, discourse analysis, ethnography, phenomenology, content analysis

The overarching design framework is ethnographic design. This methodological approach was selected as it enabled detailed, rich description to represent the participants' experience, and is commonly utilised when attempting to understand phenomena from the participant's perspective. Additionally, this framework has been utilised in previous research on food insecurity, as it reveals the structural conditions, contextual factors, habitual behaviours, competing values, and cultural understandings that impact the participants' ability to make changes suggested in the *NEST* program.

A mixed methods study was designed with quantitative (pre-post surveys) and qualitative (post-program ethnographic interviews) components. The study was also designed to align with the consolidated criteria for reporting qualitative research (COREQ) checklist (103). This study provided complementary data for methods triangulation, which enabled a more comprehensive examination of the study objectives, and assisted in confirming or problematising results and themes identified in each data set (104-106).

Participant selection

10. Sampling - How were participants selected? e.g. purposive, convenience, consecutive, snowball
A purposive sample of volunteer NEST program graduates that attended at least four NEST workshops in 2019. Participants were recruited from seven service sites located in Melbourne, Sydney, and Newcastle.

11. Method of approach - How were participants approached? e.g. face-to-face, telephone, mail, email
All participants who participated in the NEST program in April-May 2019, were notified of the opportunity to participate in the study (wave 1) by NEST Program Facilitators (OzHarvest staff). Where possible, the researcher followed up with service providers asking them to remind NEST graduates of this opportunity again in June-August (wave 2), through the distribution of posters and flyers.

12. Sample size - How many participants were in the study?
Seventeen participants were interviewed by the lead author, from a sample of program participants that attended at least four of the six program sessions. The interviews ranged from 20-60 minutes long, and due to the contingencies of interstate fieldwork, interviews occurred face-to-face (n=16) or via video call (n=1). This sample size and approach is in line with other qualitative food security studies published with 15 to 40 interviews. Participation in the study was voluntary and informed consent will be received from all participants who agree to participate in the study.

13. Non-participation - How many people refused to participate or dropped out? Reasons?
The recruitment approach was for participants to opt into the study. The seventeen respondents that said yes to be part of a semi-structured interview, all gave informed consent and completed the interview. There were no participants who subsequently refused to participate, withdrew consent or dropped out.

Setting

14. Setting of data collection - Where was the data collected? e.g. home, clinic, workplace
There were 5 settings where interviews were conducted:

1. In the common room of a social housing residence. It was a large room with lots of tables, kitchen, couches, where participants spend time when they are not in their private small rooms. Other residents were frequently walking through, so there was quite a bit of background noise. The area was chosen by the agency staff, as it was the safest place for the residents and I (the alternative was their private rooms, which we both agreed was not appropriate). The common room was also where the NEST program took place, so it was a familiar space to the participants. The setting was very challenging for sound, at one point the heating was switched on, so it was really difficult to hear.

2. At the waiting area of a food pantry. I delivered the NEST program at this agency, so I was familiar and had built good rapport with the participants. In the food pantry hall. It was a large room with the food pantry and tables set out with the food that was delivered from another food rescue organisation. Food pantry clients came in and out picking up their food for the week. There were couches and tables set up and a small kitchen space where clients could make themselves a cup of tea or coffee while they waited to select their food and be given a parcel of essentials. There was a children's play area with toys set up, so families could come and the children could play whilst their parents/caregivers collected the food. At the food pantry it looked like there weren't a lot of fresh fruit and vegetables, piles of bread (predominately white bread) available. I sat with the participants on some comfortable chairs in the corner of the room, but it was quite noisy with children playing nearby and people coming and going. I checked the recording and it worked well enough and captured the participants voice.

3. I was provided with a quiet room upstairs of the large church. The room itself was sparse with 2 chairs, a desk, and a blow heater. It had a window looking out to the other offices so it was fine to close the door to reduce the sound. The room was optimal for interviewing, quiet and I had plenty of room to set up the equipment. Plus, the desk was in the corner of the office, so we could sit comfortably on chairs facing each other, which was nice a casual opposed to sitting on either side of a desk, which may have made the participants feel nervous.
4. A coffee shop was chosen for the interview. However, the participant was running 1 hour late from work and the cafe had closed, so we sat in the car out the front of the cafe with takeaway coffees instead. It was still a very public street in front of a supermarket with lots of people around, but it may not have been wise to have conducted the interview in the car.
5. One of the participants lived interstate, so the interview was conducted via facetime. The participant and I were both in our homes.

15. Presence of non-participants - Was anyone else present besides the participants and researchers?
As there were 5 different settings with which interviews were conducted, in some of the locations there were other non-participants nearby and in others there weren't any non-participants. The setting descriptions above from the field notes provide specific details on the presence of non-participants in each setting.

16.Description of sample - What are the important characteristics of the sample? e.g. demographic data, date:
Seventeen interviews were conducted with past NEST participants within two to ten weeks of program completion (June to August 2019). The majority were male (64.7%), born in Australia (70.6%), living in social housing or rehabilitation centres (58.8%), with a household income of <$AUD 575 per week (58.8%) (Table 5). Their ages ranged from 24 to 80 years, with a mean age of 48.8 (± 16.4) years. Overall, 47% of interview participants were classified as food insecure, with 23% experiencing very low food security.

<u>Data collection</u>

17. Interview guide - Were questions, prompts, guides provided by the authors? Was it pilot tested?
Yes, a semi-structured interview guide was developed and follow up questions and prompting were allowed. The interview guide was pilot-tested with colleague and revised. The interview guide has been included as a supplementary material.

18. Repeat interviews - Were repeat interviews carried out? If yes, how many?
No.

19. Audio/visual recording - Did the research use audio or visual recording to collect the data?
The semi-structured interviews were recorded using a voice recorder and brief notes were also taken by the researcher.

20. Field notes - Were field notes made during and/or after the interview or focus group?
Yes, detailed field notes were made after each interview as part of the ethnographic framework underpinning this study.
21. Duration - What was the duration of the interviews or focus group?
The interviews duration ranged from 13:12 to 58:54 (minutes: seconds)

22. Data saturation - Was data saturation discussed?
Data saturation was considered, but the timeframe of the Masters Thesis project ended up dictating when the data collection period ended.

23. Transcripts returned - Were transcripts returned to participants for comment and/or correction?
No, but participants were provided with a summary of the study's findings.

Domain 3: Analysis and Findings

Data analysis

24. Number of data coders - How many data coders coded the data?
The first author was the primary data coder but met with second author to discuss emergent themes and coding process.

25. Description of the coding tree - Did authors provide a description of the coding tree?
Analysis involved actively identifying, coding, and classifying patterns and themes related to the study objectives, and then within each study object coding for key themes commenced.

26. Derivation of themes - Were themes identified in advance or derived from the data?
The research questions and the four dimensions of food security formed the initial framework for coding. Themes and subthemes were then created through amalgamation and separation of these initial codes.

27. Software - What software, if applicable, was used to manage the data?
The data was managed using computer assisted software (NVIVO 12 Plus), which increased the efficiency and constancy of the results.

28. Participant checking - Did participants provide feedback on the findings?
No, 2 participants responded that they were thankful to receive the summary of the study's findings.

Reporting

29. Quotations presented - Were participant quotations presented to illustrate the themes / findings? Was each quotation identified? e.g. participant number
Yes, quotations are presented to illustrated themes and each quotation was identified with a participant number 1-17.

30. Data and findings consistent - Was there consistency between the data presented and the findings?
Yes.

31. Clarity of major themes - Were major themes clearly presented in the findings?
Yes.

32. Clarity of minor themes - Is there a description of diverse cases or discussion of minor themes?
Minor and subthemes are included in the manuscript.
